# Indomethacin Increases Neurogenesis across Age Groups and Improves Delayed Probe Trial Difference Scores in Middle-Aged Rats

**DOI:** 10.3389/fnagi.2017.00280

**Published:** 2017-09-05

**Authors:** James A. McGuiness, Rachel B. Scheinert, Aditya Asokan, Vivien-Charlott Stadler, Christian S. Lee, Asha Rani, Ashok Kumar, Thomas C. Foster, Brandi K. Ormerod

**Affiliations:** ^1^Department of Neuroscience, University of Florida Gainesville, FL, United States; ^2^McKnight Brain Institute, University of Florida Gainesville, FL, United States; ^3^J. Crayton Pruitt Family Department of Biomedical Engineering, University of Florida Gainesville, FL, United States

**Keywords:** hippocampal neurogenesis, subependymal neurogenesis, learning, memory, microglia, aging, indomethacin, rosiglitazone

## Abstract

We tested whether indomethacin or rosiglitazone treatment could rejuvenate spatial ability and hippocampal neurogenesis in aging rats. Young (4 mo; *n* = 30), middle-aged (12 mo; *n* = 31), and aged (18 mo; *n* = 31) male Fischer 344 rats were trained and then tested in a rapid acquisition water maze task and then fed vehicle (500 μl strawberry milk), indomethacin (2.0 mg/ml), or rosiglitazone (8.0 mg/ml) twice daily for the remainder of the experiment. A week after drug treatment commenced, the rats were given 3 daily BrdU (50 mg/kg) injections to test whether age-related declines in neurogenesis were reversed. One week after the final BrdU injection (~2.5 weeks after the 1st water maze session), the rats were trained to a find novel hidden water maze platform location, tested on 15 min and 24 h probe trials and then killed 24 h later. During the first water maze session, young rats outperformed aged rats but all rats learned information about the hidden platform location. Middle-aged and aged rats exhibited better memory probe trial performances than young rats in the 2nd water maze session and indomethacin improved memory probe trial performances on the 2nd vs. 1st water maze session in middle-aged rats. Middle-aged rats with more new neurons had fewer phagocytic microglia and exhibited better hidden platform training trial performances on the 2nd water maze session. Regardless of age, indomethacin increased new hippocampal neuron numbers and both rosiglitazone and indomethacin increased subependymal neuroblasts/neuron densities. Taken together, our results suggest the feasibility of studying the effects of longer-term immunomodulation on age-related declines in cognition and neurogenesis.

## Introduction

Over the next two decades, 10,000 baby boomers will retire each day in the USA alone (Mather et al., 2015). The development of strategies to sustain healthy cognition across lifespan is becoming even more critical as the world's aging populations burgeon. Episodic memory for people, facts, and events across time and space requires the hippocampus and is particularly vulnerable to age in humans and in animal models of aging (Foster et al., 2012). Episodic memory impairments can emerge in middle age and increase in both incidence and severity as aging progresses (Jagust, 2013; Samson and Barnes, 2013). Hippocampal neurogenesis, which is linked to healthy cognition, declines with age across mammalian species (Kuhn et al., 1996; Eriksson et al., 1998; Cameron and McKay, 1999; Gould et al., 1999b; Bizon et al., 2004; Siwak-Tapp et al., 2007; Dupret et al., 2008; Hattiangady and Shetty, 2008; Aizawa et al., 2009) and in response to neuroinflammation (Ekdahl et al., 2003; Monje et al., 2003; Ormerod et al., 2013; Speisman et al., 2013a). Inflammatory signaling can become dysregulated with age and is a risk factor for age-related cognitive decline (Barrientos et al., 2006; Vasto et al., 2007; Villeda et al., 2011; Erickson et al., 2012; Brothers et al., 2013; Rooney, 2014; Scheinert et al., 2015; Di Benedetto et al., 2017). These data suggest that immunomodulatory strategies could preserve cognition across lifespan, perhaps through effects on neurogenesis.

Interventions that improve hippocampus-dependent cognition in aging rodents can also modulate neurogenesis and neuroinflammation. For example, exercise and exposure to an enriched environment improves spatial learning/memory measures, modulates neuroimmune signaling and increases neurogenesis in aging rodents (Kempermann et al., 1998; van Praag et al., 1999, 2005; Ziv et al., 2006; Kohman et al., 2011, 2012; Jurgens and Johnson, 2012; Kumar et al., 2012; Williamson et al., 2012; Speisman et al., 2013a,b). The broad spectrum non-steroidal anti-inflammatory drug (NSAID) indomethacin and the selective peroxisome proliferator-activated receptor-γ (PPAR-γ) activator rosiglitazone can protect both neurogenesis and cognition in young adult rodents from the effects of the immunostimulant lipopolysaccharide and γ-irradiation targeted at the hippocampus that produces an inflammatory response (Ekdahl et al., 2003; Monje et al., 2003; Ormerod et al., 2013). Indomethacin inhibits cyclooxygenase (COX)-1 and -2 activity and activates PPAR-γ, which suppresses microglial recruitment and the release of pro-inflammatory cytokines such as IL-6, TNFα, and IL-1β that can be upregulated with aging (Jiang et al., 1998; Dannhardt and Kiefer, 2001; Monje et al., 2003; Franceschi and Campisi, 2014). Our work in young rodents suggests that direct PPAR-γ activation could protect cognition and neurogenesis from the effects of age without the adverse side effects produced by COX inhibition (Goncalves et al., 2010; Ormerod et al., 2013; Pergolizzi et al., 2016).

We tested whether the broad spectrum NSAID indomethacin or the PPAR-γ activator rosiglitazone could reverse age-related declines in cognition and neurogenesis. Young, middle-aged and aged rats were trained and tested in a rapid acquisition water maze task sensitive to age-related cognitive decline and its interventions (Foster et al., 2003; Foster and Kumar, 2007; Carter et al., 2009; Speisman et al., 2013a,b; Scheinert et al., 2015) so that they could be assigned uniformly to treatment groups according to age and probe trial scores. A week after the onset of drug treatment, the rats were injected with bromodeoxyuridine (BrdU) to test whether hippocampal and subependymal neurogenesis declined with age in individual rats and could be rejuvenated by drug treatment. A week after BrdU injections, the rats were trained to locate a novel water maze platform location, tested on probe trials 15 min and 24 h later and then killed 24 h after the final probe trial to quantify neurogenesis and neuroinflammation. We hypothesized that drug treatment would improve water maze scores, increase new neuron number and reduce neuroinflammation markers in aging rats.

## Materials and methods

### Subjects

All rats were treated in accordance with federal (National Institutes of Health Publications No. 8023, revised 1978) and University of Florida Institutional Animal Care and Use Committee (IACUC) policies regarding the ethical use of animals for research. The University of Florida IACUC committee approved the animal protocols that we employed. Young (4 mo; *n* = 32), middle-aged (12 mo; *n* = 32) and aged (18 mo; *n* = 34) male Fischer 344 rats were obtained in 6 batches of 4–7 rats per age group (due to monthly order limits) from the National Institute of Aging (NIA) colony at Harlan Laboratories. Upon arrival, rats were pair-housed in corn cob bedding-lined shoebox cages in a colony room maintained at 24 ± 1°C on a 12:12 h light:dark cycle and given Harlan Teklad Rodent Diet #8604 and water *ad libitum* for the duration of the experiment. Body masses were recorded every other day to monitor potential NSAID-induced gastrointestinal side effects and general health was assessed daily. We humanely euthanized 1 middle-aged rat and 3 aged rats that exhibited age-related health problems (i.e., excessive weight loss and/or tumor growth) during the study.

**Figure 1:**
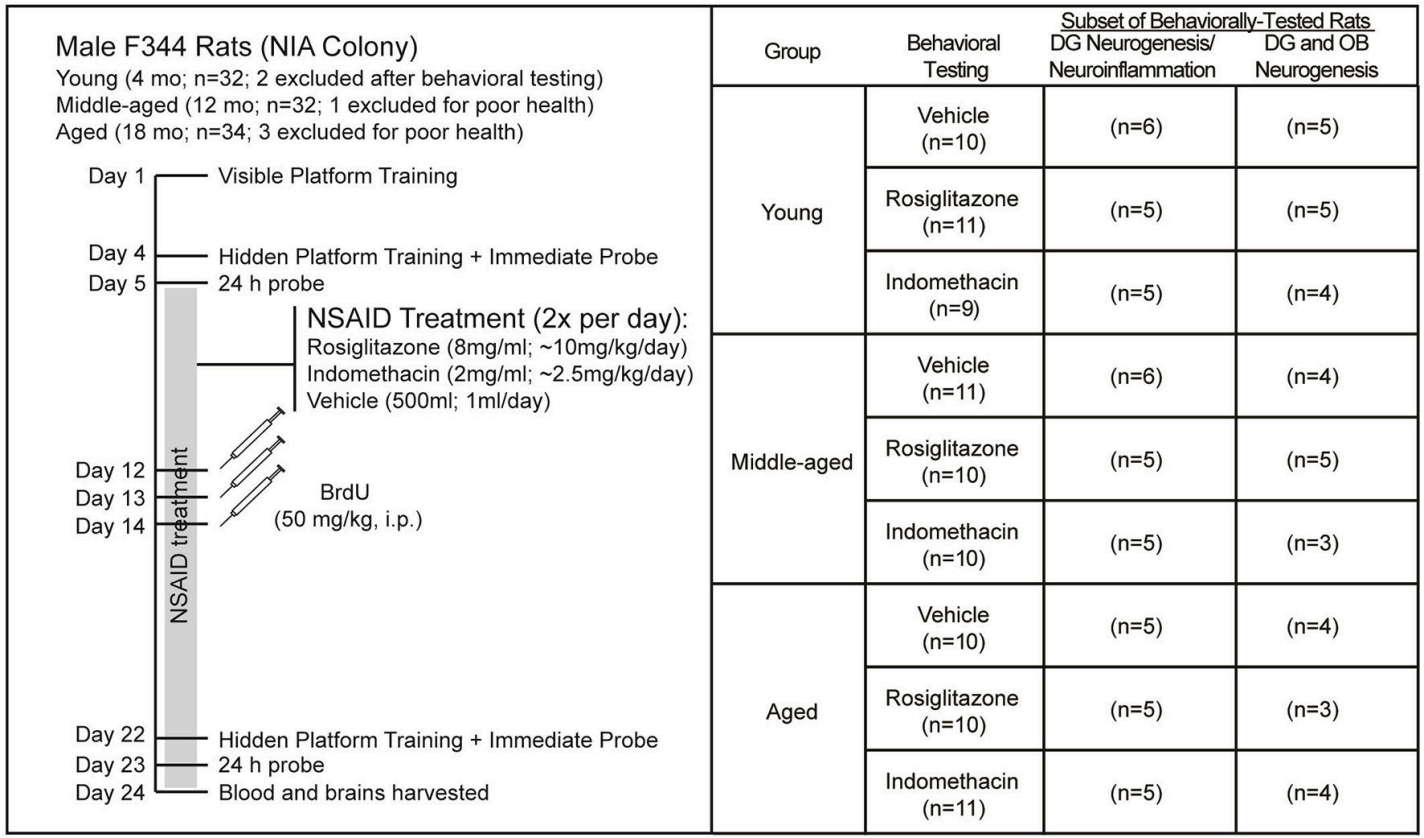
Experiment Design. Young (4 mo; *n* = 32), middle-aged (12 mo; *n* = 32), and aged (18 mo; *n* = 34) male F344 rats were trained on 5 visible platform trial blocks followed 3 d later by 4 hidden platform trial blocks and then probe trials 15 min and 24 h later. After uniform assignment to drug treatment groups, rats were fed vehicle (500 μl), indomethacin (2.0 mg/ml) or rosiglitazone (8 mg/ml) 2x a day (12 h apart; BID) until the experiment endpoint. After a week of drug treatment, rats were injected daily over 3 d with BrdU (50 kg/kg) and after 18 d of NSAID treatment, the rats were trained to locate a novel hidden platform position in a 2nd water maze session and then given probe trials 15 min and 24 h later and killed 24 h after the final probe trial. Two young rats were excluded from the study after exhibiting excessive thigmotaxia in the 1st water maze session and 1 middle-aged rat and 3 aged rats were excluded from the study because of poor health. Final numbers included in each analysis are shown. Note that subependymal and hippocampal neurogenesis was quantified in the same rats, but that a smaller subset is reported for subependymal zone neurogenesis because some sections were lost.

Figure 1 shows the experiment timeline and the number of rats obtained and included in each analysis. A week after arrival, the rats were trained to locate a visible platform and then a hidden platform 3 d later followed by immediate and 24 h delayed probe trials in the first water maze session. Two young rats were removed from the study after the first water maze session because they exhibited thigmotaxic behavior and immediate probe discrimination index (DI) scores < 0 that reflect failure to learn the spatial maze strategy. The next day, the rats were assigned uniformly by their combined probe trial DI scores (ranked 1–6 by batch) and age to treatment groups and began twice daily feedings (12 h apart) of frozen strawberry milk vehicle (500 μl), indomethacin (2.0 mg/ml) or rosiglitazone (8.0 mg/ml) treats that continued through the experiment endpoint. A week later, the rats were injected intraperitoneally (i.p.) once daily over 3 d with BrdU (50 mg/kg, i.p.) to label dividing cells. A week after the final BrdU injection, rats were trained to locate a new hidden platform position, tested on an immediate and a 24 h delayed probe trial and then killed 24 h after the final probe trial.

### Water maze training and testing

Water maze training and testing was conducted as described previously (Foster et al., 2003; Foster and Kumar, 2007; Carter et al., 2009; Speisman et al., 2013a,b; Scheinert et al., 2015) in a well-lit room containing a black water maze (1.7 m d) filled with water (27 ± 2°C) such that the water surface was ~8–10 cm below the tank rim. A platform (13 cm d) protruded 1.5 cm above the water surface on visible platform trials or was submerged 1.5 cm below the water surface on hidden platform trials. Swim times, distances, and speeds were recorded with a Columbus Instruments tracking system. Rats were towel dried and warm air was blown over their holding cages between trial blocks.

#### Visible platform trials

Rats were initially released from random pool locations, guided gently to the platform and then removed 30 s later on 4 unrecorded habituation trials. Immediately afterward, latencies (s) and pathlengths (cm) were recorded and swim speeds (cm/s) calculated as measures of sensorimotor ability and motivation on 5 blocks (15 min inter-block interval) of 3 60-s visible platform trials (20 s inter-trial interval). A black curtain surrounding the maze masked extramaze cues and a large white flag was affixed to the platform. Platform locations and release points were randomized across trials and rats were guided gently to the platform after 60 s.

#### Hidden platform trials

Latencies (s) and pathlengths (cm) were recorded as measures of spatial ability and swim speeds were calculated as measures of sensorimotor ability and motivation on hidden platform trials conducted in the presence of highly visible extramaze cues 3 d after visible platform training. Rats were trained on 4 blocks (15 min inter-block interval) of three 60-s trials (20 s inter-trial interval) to locate the platform hidden in the NE water maze quadrant. Note that the number of hidden platform training blocks was reduced from the 5 that we typically administer (Foster et al., 2003; Foster and Kumar, 2007; Carter et al., 2009; Speisman et al., 2013a,b; Scheinert et al., 2015) to reduce probability that memory for the platform location learned in the 1st water maze session (before drug treatment) would interfere with learning the novel platform location ~2.5 weeks later during the 2nd water maze session (during drug treatment), particularly since we administered all training blocks in a single session. Training was identical in both water maze sessions, except that the platform was hidden in SE water maze quadrant in the 2nd session. On each trial, rats were released from one of four randomized start locations and given 60 s to locate and escape onto the platform before being guided.

#### Probe trials

Discrimination Index (DI) scores and % time spent in the goal quadrant served as respective strength of learning and memory measures in probe trials administered 15 min and 24 h after hidden platform training in each session. DI scores (*t*(G) − *t*(O))/(*t*(G) + *t*(O)), where *t*(G) and *t*(O) reflect time spent in the goal and opposite water maze quadrants) produce higher fidelity memory indices for aged rats that swim slower and make more sweeping turns than young rats. Note that a DI score of 0.33 confirms 2x as much time spent in the goal vs. opposite quadrant and a DI score that approaches 1 confirms that nearly all of the time was spent in the goal vs. opposite quadrant on a 60 s probe trial. Rats were released from opposite quadrant and given 60 s to swim in absence of the platform.

### Non-steroidal anti-inflammatory drug treatment

All rats voluntarily consumed frozen Nestle Nesquick Strawberry milk™ vehicle treats (500 μl) 2x per day in their home cages before and during the first water maze session (~7 days). Beginning 24 h after the first water maze session, rats voluntarily consumed frozen vehicle (500 μl), indomethacin (2.0 mg/ml; Sigma Aldrich, St. Louis, MO, USA) or rosiglitazone (8.0 mg/ml; Cayman Chemical Company, Ann Arbor, MI, USA) treats 2x per day (12 h apart) in individual feeding cages according to their drug treatment group assignment (Figure 1): vehicle (*n* = 10 young, *n* = 11 middle-aged, and *n* = 10 aged), indomethacin (*n* = 9 young, *n* = 10 middle-aged, and *n* = 11 aged) and rosiglitazone (*n* = 11 young, *n* = 10 middle-aged, and *n* = 10 aged). Each rat was returned to its home cage immediately after the entire frozen vehicle, indomethacin or rosiglitazone treat was consumed. Drug treatment continued throughout the duration of the experiment (Days 5–24; 20 days).

Strawberry milk treats were prepared as described previously (Ormerod et al., 2013). Briefly, ethanol was added to Nestle Nesquick Strawberry milk™ at a concentration of 50 μl/ml to produce vehicle. Powdered indomethacin (2.0 mg) and crushed rosiglitazone (8.0 mg) was dissolved in 50 μl of ethanol and the drug ethanol solution was added to strawberry milk at a concentration of 50 μl/ml. Each milk solution was prepared at the beginning of the experiment and frozen in 500 μl aliquots as treats in small plastic petri dishes stored at −20°C. Given our assumption that the ~400 g body mass recorded in the first batch of rats received would remain consistent across subsequent batches, these concentrations approximated clinical indomethacin (2.5 mg/kg/day) and rosiglitazone (10 mg/kg/day) dosages (Monje et al., 2003; Norris et al., 2007) and produced a 0.005 g/kg ethanol dosage that neither impacts neurogenesis nor spatial behavior (Nixon and Crews, 2002; Crews et al., 2006; Ormerod et al., 2013). Final mean (±*S.E.M*.) body masses were 354.5 ± 33.0 g in young, 463.6 ± 24.0 g in middle-aged, and 454.1 ± 34.1 g in aged rats making final indomethacin dosages range from 2.6 to 3.1 mg/kg in young, 2.1 to 2.3 mg/kg in middle-aged, and 2.1 to 2.4 mg/kg in aged rats and rosiglitazone dosages range from 10.4 to 15.2 mg/kg in young, 8.1 to 9.2 mg/kg in middle-aged and 8.1 to 9.6 mg/kg in aged rats, which were all within clinical ranges.

### Bromodeoxyuridine injections

Rats were injected i.p. with BrdU (Sigma Aldrich) daily over 3 d beginning a week after the onset of drug treatment, which was a week after the 1st water maze session and a week before the 2nd water maze session to minimize the effects of spatial behavior on neural progenitor cell (NPC) proliferation and new neuron survival (Gould et al., 1999a; Epp et al., 2010). BrdU was dissolved in freshly prepared 0.9% sterile saline at a concentration of 20 mg/mL and injected at a volume of 2.5 ml/kg (50 mg/kg).

### Histology

The day after the final probe trial in the second water maze session, rats were anesthetized with isoflurane (Halocarbon Laboratories, River Edge, NJ) and decapitated. Serum supernatant harvested from trunk blood was refrigerated for 2 h at 4°C, centrifuged at 1,000 × g for 10 min and then stored at −86°C for future analysis. Brains were extracted and dissected laterally. Hippocampi dissected from left side of the brain were flash frozen and stored at −86°C for future work. The right side of the brain was post-fixed overnight in fresh 4% paraformaldehyde (Electron Microscopy Sciences; Hatfield, PA) and then equilibrated in 30% sucrose (~4 days) at 4°C. Postfixed ½ brains were sectioned coronally at 40 μm intervals on a freezing stage sledge microtome (Model 860; American Optical Corporation; IMEB Inc., San Marcos, CA). Sets of every six sections through the subependymal zone including the rostral migratory stream (RMS) and olfactory bulb (OB) and through the hippocampal dentate gyrus were stored at −20°C in cryoprotectant solution (30% ethylene glycol, 25% glycerin, and 45% 0.1 M PBS).

### Immunohistochemistry

Immunohistochemistry was conducted as described previously (Bruijnzeel et al., 2011; Chen et al., 2011; Lee et al., 2013; Ormerod et al., 2013; Asokan et al., 2014) on free floating sections washed repeatedly between steps with tris-buffered saline (TBS; pH 7.4). Blocking and antibody solution was 3% normal donkey serum and 0.1% Triton-X in TBS. BrdU^+^ cells and ionized calcium binding adaptor molecule 1 (Iba1)^+^ microglia were revealed on separate sets of dentate gyrus sections and BrdU^+^ cells were revealed on RMS and OB sections using 3,3′-diaminobenzidine tetrahydrochloride (DAB) to calculate total cell numbers and densities under light microscopy. BrdU^+^ cell and Iba1^+^ microglia phenotypes were confirmed on separate sets of sections co-labeled with phenotypic makers revealed by fluorophore-conjugated secondary antibodies under confocal microscopy.

#### Enzyme substrate immunostaining

Sections were incubated in 0.3% H_2_O_2_ in TBS for 10 min to quench endogenous peroxidase and then blocking solution for 20 min. Sections immunostained to detect BrdU were rinsed in 0.9% NaCl, incubated in 2N HCl for 20 min at 37°C to denature DNA, overnight in either rat anti-BrdU (1:500; AbD Serotec, Raleigh, NC) or rabbit anti-Iba1 (1:1,000; Wako, Osaka, Japan) at 4°C and then biotinylated secondary anti-rat IgG and biotinylated anti-rabbit IgG, respectively (Jackson ImmunoResearch, West Grove, PA; 1:500) for 4 h at room temperature (RT). Finally, sections were incubated in avidin-biotin horseradish peroxidase (Vector Laboratories, Burlingame, CA) and then reacted in a solution of 0.02% DAB (Sigma Aldrich, St. Louis, MO) and 0.5% H_2_O_2_ before being mounted on glass slides, dried overnight, dehydrated in an alcohol series and cover-slipped under permount (Fisher Scientific).

#### Fluorescent immunostaining

Sections were blocked for 20 min at RT and then incubated overnight in primary antibodies raised against (1) the immature neuronal protein doublecortin (goat anti-DCX, 1:500; Santa Cruz Biotechnology, Santa Cruz, CA) and the mature neuronal protein neuronal nuclei (mouse anti-NeuN, 1:500; Chemicon, Temecula, CA), (2) the astrocyte protein glial fibrillary acidic protein (chicken anti-GFAP, 1:500; Encor Biotech, Alachua, FL) and the oligodendrocyte protein chondroitin sulfate proteoglycan (rabbit anti-NG2; Chemicon, 1:500; Temecula, CA) or (3) the microglial protein Iba1 (rabbit anti-Iba1, 1:1,000; Wako, Osaka, Japan), the activation marker CD11b (mouse anti-CD11b, 1:500; Millipore, Billerica, MA) and the phagocytic marker CD68 (goat anti-CD68, 1:500; Santa Cruz Biotechnology, Santa Cruz, CA) at 4°C and then incubated in maximally cross-adsorbed fluorophore-conjugated secondary antibodies (1:500; Jackson ImmunoResearch, West Grove, PA) for 4 h at RT. The sections immunostained for neuronal and glial markers were then rinsed in 0.9% NaCl, incubated in 2N HCl at 37°C for 20 min, blocked for 20 min at RT, incubated in rat anti-BrdU (1:500; AbD Serotec, Raleigh, NC) overnight at 4°C and then incubated in maximally cross-adsorbed fluorophore-conjugated anti-rat secondary antibody (1:500; Jackson ImmunoResearch, West Grove, PA) for 4 h at RT. All sections were counterstained with 4′,6-diamidine-2′-phenylindole (DAPI; Calbiochem, San Diego, CA; 1:10,000) for 10 min at RT and mounted under diazobicyclooctane (DABCO; 2.5% DABCO, 10% polyvinyl alcohol, and 20% glycerol in TBS).

### Quantification of neurogenesis and microglia

#### Total new cell and microglial cell numbers and densities

BrdU^+^ cells and Iba1^+^ microglia were counted exhaustively through the dentate gyrus on separate sets of every 6th (240 μm apart; 12 sections/rat) and every 12th (480 μm apart; 6 sections/rat) systemically uniform DAB-stained section, respectively through the rostral-caudal extent of the dentate gyrus under a 40x objective on a Zeiss Axio Observer Z1 inverted microscope (Carl Zeiss, Thornwood, NY, USA). The first section of each set counted was randomly chosen from sections taken between −1.72 and −1.92 A/P to bregma (Paxinos and Watson, 2007). Total BrdU^+^ and Iba1^+^ cell numbers in the dentate gyrus were estimated using optical fractionator principles (West et al., 1991; Kempermann et al., 2002; Boyce et al., 2010; Speisman et al., 2013b). BrdU^+^ and Iba1^+^ cell counts were multiplied by 6 and 12, respectively to account for the section interval and by 2 to account for use of ½ of the brain. Areas where BrdU^+^ cells (subgranular zone and granule cell layer) and Iba1^+^ microglia (granule cell layer and hilus) were counted were measured under a 10x objective using MicroBrightfield StereoInvestigator software (Williston, VT, USA) to calculate cell densities for comparison with OB measures. In addition, dentate gyrus volumes were estimated using Cavalieri's principle (Uylings et al., 1986; Galea et al., 2000; Seifert et al., 2010): *Volume* = $\underset{\text{sections}}{\sum }$ × ¼ *I (h1* + √*h1* × √*h2* + *h2)* where *I* is the interval between sections, and *h1* and *h2* are two sections between which the volume is estimated.

BrdU^+^ cells were counted in the vertical limb of the RMS (RMS_VL_; between the lateral ventricle and elbow), the horizontal limb of the RMS (RMS_HL_; between the elbow and OB, rostral migratory stream of the OB (RMS_OB_; the remainder of the RMS within the OB), and the OB granule cell layer (GCL_OB_) on every 6th section between +2.76 and +7.56 A/P to bregma (Paxinos and Watson, 2007). Areas of the regions that the cells were counted upon were measured using MicroBrightfield StereoInvestigator software. Because we did not obtain systematically uniform sections through each RMS and OB region, densities rather than total cell estimates are reported.

#### Microglial and new cell and phenotypes

On fluorophore-stained sections, the resting, activated and phagocytic phenotypes of at least 100 Iba1^+^ microglia (~2 sections per rat) and the neuronal or glial phenotypes of at least 100 BrdU^+^ hippocampal cells (~6 sections per rat) and 100 BrdU^+^ olfactory bulb cells (~6 sections per rat) was confirmed under a 20x objective with 1.5x digital zoom on a Zeiss LSM 710 meta confocal microscope with 405, 488, 510, 543, and 633 laser lines (Carl Zeiss, Thornwood, NY, USA). BrdU^+^/DAPI^+^ nucleii were scanned through their full z-planes to reveal whether they clearly expressed (1) DCX and/or NeuN (new, transitioning or mature neurons, respectively), (2) NG2, or (3) GFAP. The percentage of BrdU^+^ cells expressing each phenotype was calculated. DAPI/Iba1^+^ nuclei were scanned through their full z-planes to reveal whether they clearly expressed the activation marker CD11b and/or the phagocytic marker CD68 (Prasad et al., 2012) and the percentage of Iba1^+^ microglia expressing each phenotype was calculated.

### Statistical analyses

All statistical analyses were conducted with Version 13 STATISTICA software (http://onthehub.com/). Analyses of variance (ANOVA) explored the effects of the independent variables (age and drug treatment) on water maze performances (latencies, pathlengths and swim speeds across trial blocks, % time spent in goal and opposite quadrants), BrdU^+^ cells (total numbers, percentages of new neurons and glia and total numbers of new neurons and glia) and Iba1^+^ microglia (total numbers, percentages of resting, activated, and phagocytic microglia and total numbers of resting, activated, and phagocytic microglia) and group differences were revealed by Newman Keuls *post-hoc* tests. Kruskal–Wallis ANOVAs tested the effects of the independent variables (age and drug treatment) on non-continuous behavioral variables (DI scores and difference scores) and group differences were revealed by Mann–Whitney *U*-tests. Dependent *t*-tests confirmed differences in performance across binned hidden platform training blocks (1st and 2nd combined vs. 3rd and 4th combined) and Pearson Product Moment correlation coefficient analyses tested the strength of relationships between variables. All data are shown as group means (± S.E.M.) or as individual probe trial DI scores. The α-level was set at *p* < 0.05.

## Results

### Rats rapidly learned but did not remember a hidden platform location

#### All rats located a visible platform

**Figure 2:**
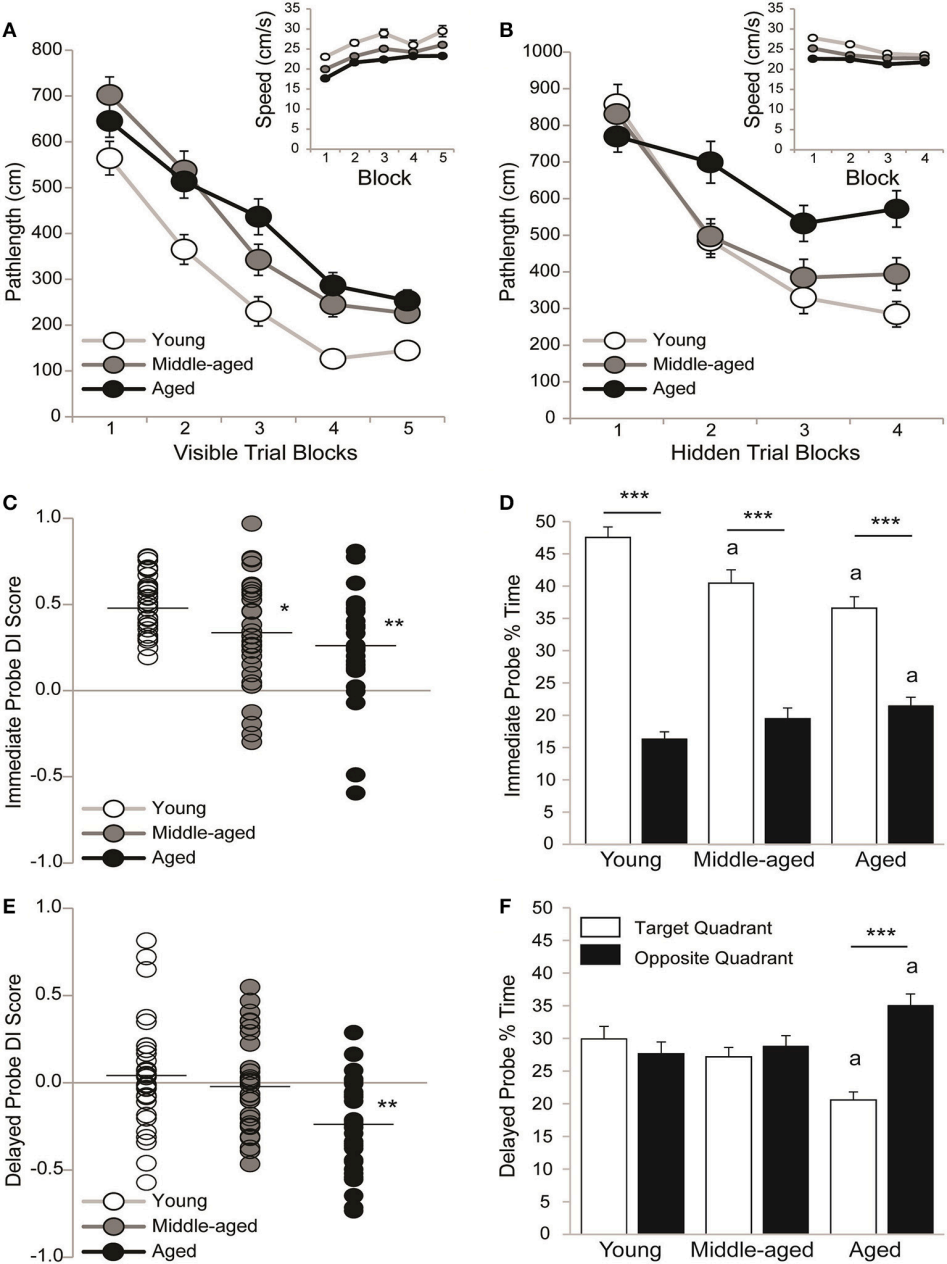
All rats learned information about but did not remember a hidden platform location. Group means (±S.E.M.) or individual DI scores with group means represented by the black line are shown for young (white circles), middle-aged (gray circles), and aged (black circles) rats and % time spent in the target (white bar) and opposite (black bar) water maze quadrant. **(A)** Rats swam more directly to a visible platform across training blocks (Block 1 > 2 > 3 > 4 and 5; *p* < 0.0001) but young rats outperformed middle-aged and aged rats (*p* < 0.001) on all blocks combined. Swim speeds declined with age (inset; young vs. middle-aged vs. aged; *p* < 0.05). **(B)** Aged rats swam more circuitous routes to the hidden platform that young or middle-aged rats (*p* < 0.01) and young rats showed more uniform improvement across training blocks (Block 1 > 2 > 3 and 4; *p* < 0.05) than middle-aged (Block 1 > 2–4; *p* < 0.001) and aged rats (Block 1,2>3,4; *p* < 0.05). Swim speeds declined with age (young vs. middle-aged vs. aged; *p* < 0.01). **(C)** Young rats outperformed middle-aged (^*^p < 0.05) and aged rats (^**^p < 0.01) on the immediate probe trial. **(D)** During the immediate probe trial, all age groups spent more time in the target vs. opposite quadrant (^***^p < 0.001) but middle-aged and aged rats spent less time in the target quadrant (^a^*p* < 0.05) and aged rats spent more time in the opposite quadrant (^a^*p* < 0.01) than young rats. **(E)** DI scores were lower in aged rats than in young or middle-aged rats (^**^p < 0.01). **(F)** Aged rats spent more time in the opposite vs. target quadrant (^***^p < 0.001) and less time in the target and more time in the opposite quadrant than young rats (^a^*p* < 0.05) but neither young nor middle-aged rats discriminated the target quadrant.

Sensorimotor ability and motivation to escape the pool was tested on visible platform trials. Pathlengths are reported because young rats swim faster than aging rats; however pathlengths correlated with latencies across visible (*all r* ≥ 0.88 and *p* < 0.01) and hidden (*all r* ≥ 0.91 and *p* < 0.01) trial blocks. Figure 2A shows that pathlengths varied by age [*F*_(2, 89)_ = 21.61; *p* < 0.0001] and trial block [*F*_(4, 356)_ = 117.09; *p* < 0.001] but not the overall interaction [*F*_(8, 356)_ = 1.46; *p* = 0.17]. Although young rats swam more direct pathlengths than middle-aged (*p* < 0.001) and aged (*p* < 0.001) rats, pathlengths generally decreased as training progressed (Block 1 > 2 > 3 > 4 and 5; *p* < 0.0001). The inset shows that swim speeds across trial blocks varied by age [*F*_(2, 89)_ = 19.63; *p* < 0.001] and trial block [*F*_(4, 356)_ = 12.53; *p* < 0.001] but not the overall interaction [*F*_(8, 356)_ = 0.19; *p* = 0.99]. Young rats swam faster than middle-aged (*p* < 0.001) and aged (*p* < 0.001) rats and middle-aged rats swam faster than aged rats (*p* < 0.05). Generally, swim speeds increased with training (Block 1 > 2-5; *p* < 0.001).

#### All rats learned the hidden platform location

Spatial ability was tested on hidden platform trial blocks. Figure 2B shows that pathlengths varied by age [*F*_(2, 89)_ = 7.08; *p* < 0.01], trial block [*F*_(3, 267)_ = 59.02; *p* < 0.0001] and the overall interaction [*F*_(6, 267)_ = 3.90; *p* = 0.001]. Regardless of age, pathlengths decreased as training progressed (Block 1 > 2 > 3, and 4; *p* < 0.001) but aged rats swam more circuitous routes than either young (*p* < 0.01) or middle-aged rats (*p* < 0.01). More specifically, young rats outperformed aged rats on Blocks 2 (*p* < 0.05), 3 (*p* < 0.05) and 4 (*p* < 0.001) and middle-aged rats outperformed aged rats on Block 2 (*p* < 0.05). Moreover, pathlengths decreased steadily across blocks in young rats (Block 1 > 2 ≥ 3 and 4; *p* < 0.05) but early before plateauing in middle-aged rats (Block 1 > 2–4; *p* < 0.001) and later before plateauing in aged rats (Blocks 1 and 2 > 3 and 4; *p* < 0.05). Dependent *t*-tests confirmed that young [*t*_(29)_ = 7.83; *p* < 0.0001], middle-aged [*t*_(30)_ = 6.96; *p* < 0.0001] and aged rats [*t*_(30)_ = 4.62; *p* < 0.0001] learned information about the platform location by swimming shorter pathlengths on Trial Blocks 3 and 4 vs. 1 and 2 binned.

The Figure 2B inset shows that swim speeds varied by age [*F*_(2, 89)_ = 19.09; *p* < 0.0001], trial block [*F*_(3, 267)_ = 15.95; *p* < 0.0001] and the overall interaction [*F*_(6, 267)_ = 2.13; *p* = 0.05]. Young rats swam faster than middle-aged (*p* < 0.001) and aged (*p* < 0.001) rats and middle-aged rats swam faster than aged rats (*p* < 0.01). More specifically, young rats swam faster than middle-aged and aged rats on Blocks 1 (*p* < 0.01) and 2 (*p* < 0.01) and middle-aged rats swam faster than aged rats on Block 1 (*p* < 0.05). Generally swim speeds decreased across trial blocks (Block 1 > 2 > 3, and 4; *p* < 0.01), primarily because young rats swam more slowly as training progressed (Block 1 > 2 > 3 and 4; *p* < 0.05). Middle-aged rats swam more slowly after the first block (Block 1 > 2-4; *p* < 0.01) and aged rats exhibited consistent swim speeds across blocks.

#### All rats discriminated the hidden platform location immediately after training

Strength of learning for the hidden platform location was tested on an immediate probe trial. Figure 2C shows that all young (0.51 ± 0.03) and the majority of middle-aged (0.34 ± 0.06) and aged (0.25 ± 0.05) rats learned the hidden platform location. However, there was an effect of age [*H*_(2, 92)_ = 14.51; *p* < 0.001], such that young rats exhibited higher DI scores than either middle-aged (*p* < 0.01^*^) or aged rats (*p* < 0.0001^**^). Figure 2D shows that the % of time spent in the target and opposite pool quadrant varied by age [*F*_(2, 89)_ = 5.16; *p* < 0.01], quadrant [*F*_(1, 89)_ = 183.06; *p* < 0.0001] and the overall interaction [*F*_(2, 89)_ = 9.04; *p* < 0.001]. Young rats spent more time than middle-aged (*p* < 0.001^a^) or aged (*p* < 0.0001^a^) rats in the training quadrant and less time than aged rats (*p* < 0.05^a^) in the opposite quadrant. Regardless, young, middle-aged and aged (all *p* < 0.001^***^) rats all spent more time in the target vs. opposite quadrant.

#### Regardless of age, rats did not discriminate the training quadrant on a delayed probe trial

Memory for the hidden platform location was tested on a 24h delayed probe trial. Figure 2E shows the DI scores of young (0.05 ± 0.06), middle-aged (−0.02 ± 0.05) and aged (−0.25 ± 0.05) rats. About ½ of the young and middle-aged rats and only a few of the aged rats recalled the hidden platform location. DI scores varied by age [*H*_(2, 92)_ = 14.53; *p* < 0.001], such that aged rats exhibited lower DI scores than either young (*p* < 0.001) or middle-aged (*p* < 0.01) rats. Figure 2F shows that the % of time spent in the training and opposite quadrant varied by quadrant [*F*_(1, 89)_ = 6.01; *p* < 0.05] and the overall interaction [*F*_(2, 89)_ = 8.62; *p* < 0.001] but not by age [*F*_(2, 89)_ = 0.54; *p* = 0.58]. Aged rats spent less time in the target quadrant and more time in the opposite quadrant than either young (both *p* < 0.01^a^) or middle-aged (both *p* < 0.05^a^) rats. However, neither young (*p* = 0.76) nor middle-aged (*p* = 0.60) rats discriminated the training quadrant and aged rats spent more time in the opposite vs. training quadrant (*p* < 0.001^***^).

### Indomethacin potentiated the effect of retraining on memory in middle-aged rats

#### All rats learned a novel hidden platform location

**Figure 3:**
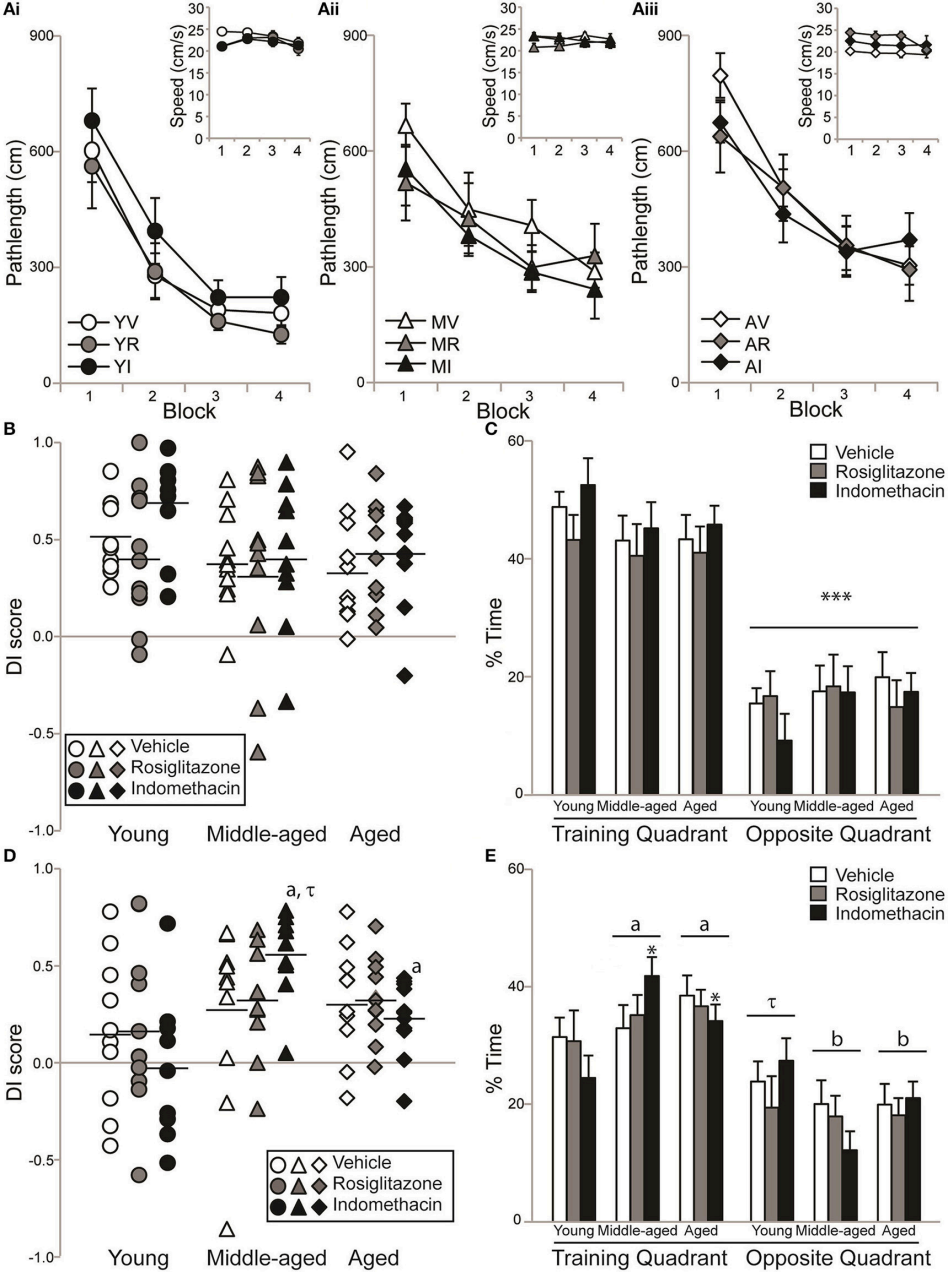
Aging rats remembered a novel platform position. Individual DI scores with means represented by the black lines and group means (±S.E.M.) are shown for young (circles), middle-aged (triangles), and aged rats (diamond) treated with vehicle (white), rosiglitazone (grey) or indomethacin (black). **(A)** Pathlengths exhibited by young **(Ai)**, middle-aged **(Aii)**, and aged **(Aiii)** rats combined decreased across training blocks (*p* < 0.0001) and young rats swam shorter distances to the platform than middle-aged (*p* < 0.05) and aged (*p* < 0.01) rats on all blocks combined. Overall, swim speeds decreased on the final block (inset; *p* < 0.01). **(B)** DI scores on the immediate probe trial did not vary by group (*p* = 0.38). **(C)** All groups spent a greater % of time in the training vs. opposite quadrant on the immediate probe trial (^***^p < 0.001). **(D)** Middle-aged and aged indomethacin-treated rats had better DI scores on the delayed probe trial than young indomethacin-treated rats (^*^p < 0.05) on the delayed probe trial. Middle-aged indomethacin-treated rats tended to outperform middle-aged vehicle-treated rats (*p* = 0.07τ). **(E)** Overall, rats spent more time in the training vs. opposite quadrant (*p* < 0.001) and middle-aged (^a^*p* < 0.05) and aged (^a^*p* < 0.05) rats spent more time in the training quadrant than young rats. Young rats only tended to spend more time in the training quadrant (^τ^*p* < 0.07) that middle-aged (^b^*p* < 0.001) and aged (^b^*p* < 0.001) rats readily discriminated.

Figure 3A shows pathlengths and the insets show swim speeds exhibited by young (Ai), middle-aged (Aii), and aged (Aiii) rats on hidden platform training trials administered during drug treatment. Pathlength varied by age [*F*_(2, 83)_ = 5.36; *p* < 0.01] and training block [*F*_(3, 249)_ = 77.21; *p* < 0.0001], but not by treatment or other interactions between the independent variables (all *p* ≥ 0.21). Pathlengths generally decreased with training (Block 1 > 2 > 3 and 4; *p* < 0.0001) but young rats swam more direct pathlengths than either middle-aged *p* < 0.05) or aged (*p* < 0.01) rats. However, dependent *t*-tests confirmed that young vehicle- [*t*_(9)_ = 5.39; *p* < 0.001], indomethacin- [*t*_(8)_ = 6.69; *p* < 0.001] and rosiglitazone- [*t*_(10)_ = 3.61; *p* < 0.01] treated rats, middle-aged vehicle- [*t*_(10)_ = 3.98; *p* < 0.01] and indomethacin- [*t*_(9)_ = 3.49; *p* < 0.01] treated rats and aged vehicle- [*t*_(9)_ = 6.49; *p* < 0.001], indomethacin- [*t*_(10)_ = 3.74; *p* < 0.01] and rosiglitazone- [*t*_(9)_ = 3.51; *p* < 0.01] treated rats swam shorter pathlengths on Trial Blocks 3 and 4 binned vs. 1 and 2 binned. This difference only approached statistical significance for middle-aged rosiglitazone-treated rats [*t*_(9)_ = 2.15; *p* = 0.06], because they exhibited good performances on the first trial block.

Swim speeds were affected by trial block [*F*_(3, 249)_ = 3.56; *p* < 0.05] and the interaction between age and treatment [*F*_(4, 83)_ = 3.29; *p* < 0.05], but not age, treatment or other interactions between the independent variables (all *p* ≥ 0.022). Generally, swim speeds were slower on the last vs. all other blocks (all *p* < 0.05) and aged vehicle-treated rats swam more slowly than young vehicle-treated rats on the first block (*p* < 0.05).

#### All rats discriminated the goal quadrant immediately after retraining

Figure 3B and mean (±S.E.M.) immediate probe trial DI scores showed that the majority of young vehicle- (0.52 ± 0.06), rosiglitazone- (0.42 ± 0.10), and indomethacin- (0.68 ± 0.08) treated, middle-aged vehicle- (0.40 ± 0.08), rosiglitazone- (0.34 ± 0.16) and indomethacin- (0.42 ± 0.12) treated and aged vehicle- (0.36 ± 0.09), indomethacin- (0.43 ± 0.08) and rosiglitazone- (0.43 ± 0.08) treated rats learned the novel platform location. In fact, DI scores confirmed consistently strong discrimination across groups [*H*_(8, 92)_ = 8.53; *p* = 0.38]. Figure 3C shows that all rats spent significantly more time in the training vs. opposite quadrant [*F*_(1, 83)_ = 168.35; *p* < 0.0001^***^] and consistent percentages across age, treatment and interactions between independent variables (all *p* ≥ 0.09).

#### Aging but not young rats remembered a novel platform position

Figure 3D and mean (±S.E.M.) 24 h delayed probe trial DI scores showed that a little over ½ of the young vehicle- (0.16 ± 0.13), rosiglitazone- (0.17 ± 0.13), and indomethacin- (−0.03 ± 0.13) treated but the majority of middle-aged vehicle- (0.27 ± 0.14), rosiglitazone- (0.33 ± 0.09) and indomethacin- (0.54 ± 0.07) treated and aged vehicle- (0.32 ± 0.09), indomethacin- (0.33 ± 0.07), and rosiglitazone- (0.23 ± 0.06) treated rats remembered the novel platform location. DI scores varied by group [*H*_(8, 82)_ = 16.59; *p* < 0.05]. Within treatment, middle-aged (*p* < 0.01^a^) and aged (*p* < 0.05^a^) indomethacin-treated rats outperformed young indomethacin-treated rats and within age, middle-aged indomethacin-treated rats tended to outperform middle-aged vehicle-treated rats (*p* = 0.07^τ^).

Figure 3E shows that % of time spent in the target and opposite quadrant varied by quadrant [*F*_(1, 83)_ = 49.28; *p* < 0.0001] and the interaction between age and quadrant [*F*_(2, 83)_ = 4.27; *p* < 0.05], but not by age, treatment or other interactions between the independent variables (all *p* ≥ 0.19). Overall, rats spent more time in the training vs. opposite water maze quadrant (*p* < 0.001) and middle-aged (*p* < 0.05^a^) and aged (*p* < 0.05^a^) rats spent more time in the training quadrant than young rats. Young rats only tended to spend more time in the training quadrant (*p* < 0.07^τ^) that middle-aged (*p* < 0.001^b^) and aged (*p* < 0.001^b^) rats readily discriminated.

#### Indomethacin improved memory probe difference scores in middle-aged rats

**Figure 4:**
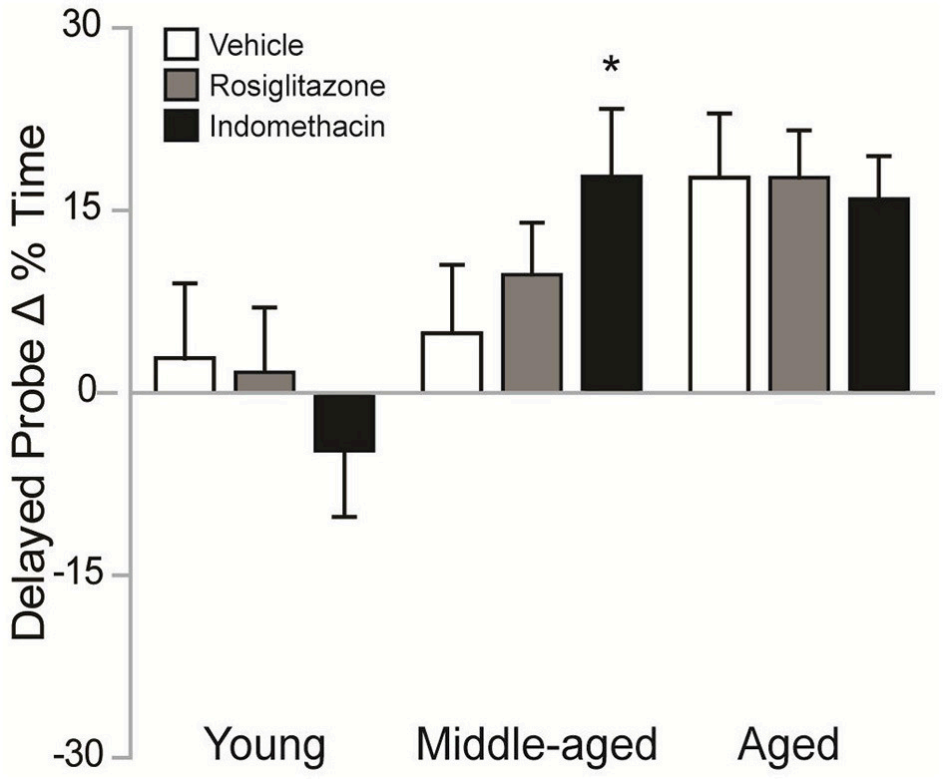
Indomethacin improved difference scores in middle-aged rats. Mean (± S.E.M.) differences in % Time spent in the target quadrant on the 2nd vs. 1st session are shown for rats treated with vehicle (white), rosiglitazone (gray), or indomethacin (black). Middle-aged indomethacin-treated rats exhibited bigger improvements in the % time spent in the target quadrant across sessions than middle-aged vehicle-treated rats (^*^p < 0.05). Although, rosiglitazone-treated aged rats (*p* < 0.05) and indomethacin-treated middle-aged and aged rats (*p* < 0.01) exhibited higher difference scores than young rats within each drug group, the unexpectedly poor delayed probe trial performances exhibited by young rats made interpretation of the effects of age within drug treatment difficult.

Figure 4 shows the difference between the % of time spent in the target quadrant on delayed probe trials in the 2nd vs. 1st water maze session. Difference scores varied by group [*H*_(8, 92)_ = 18.26; *p* < 0.05]. Although, higher difference scores were exhibited by aged vs. young rosiglitazone-treated rats (*p* < 0.05) and by middle-aged and aged vs. young indomethacin-treated rats (*p* < 0.05 and *p* < 0.01, respectively), the poor trial performances exhibited by young rats on the 2nd water maze session render the reliability and validity of the effect of age within drug treatment questionable. However, middle-aged rats exhibited better difference scores after indomethacin vs. vehicle treatment (*p* < 0.05^*^), suggesting that indomethacin potentiated the intersession improvements exhibited by middle-aged rats.

### Indomethacin increased hippocampal neurogenesis across age groups

**Figure 5:**
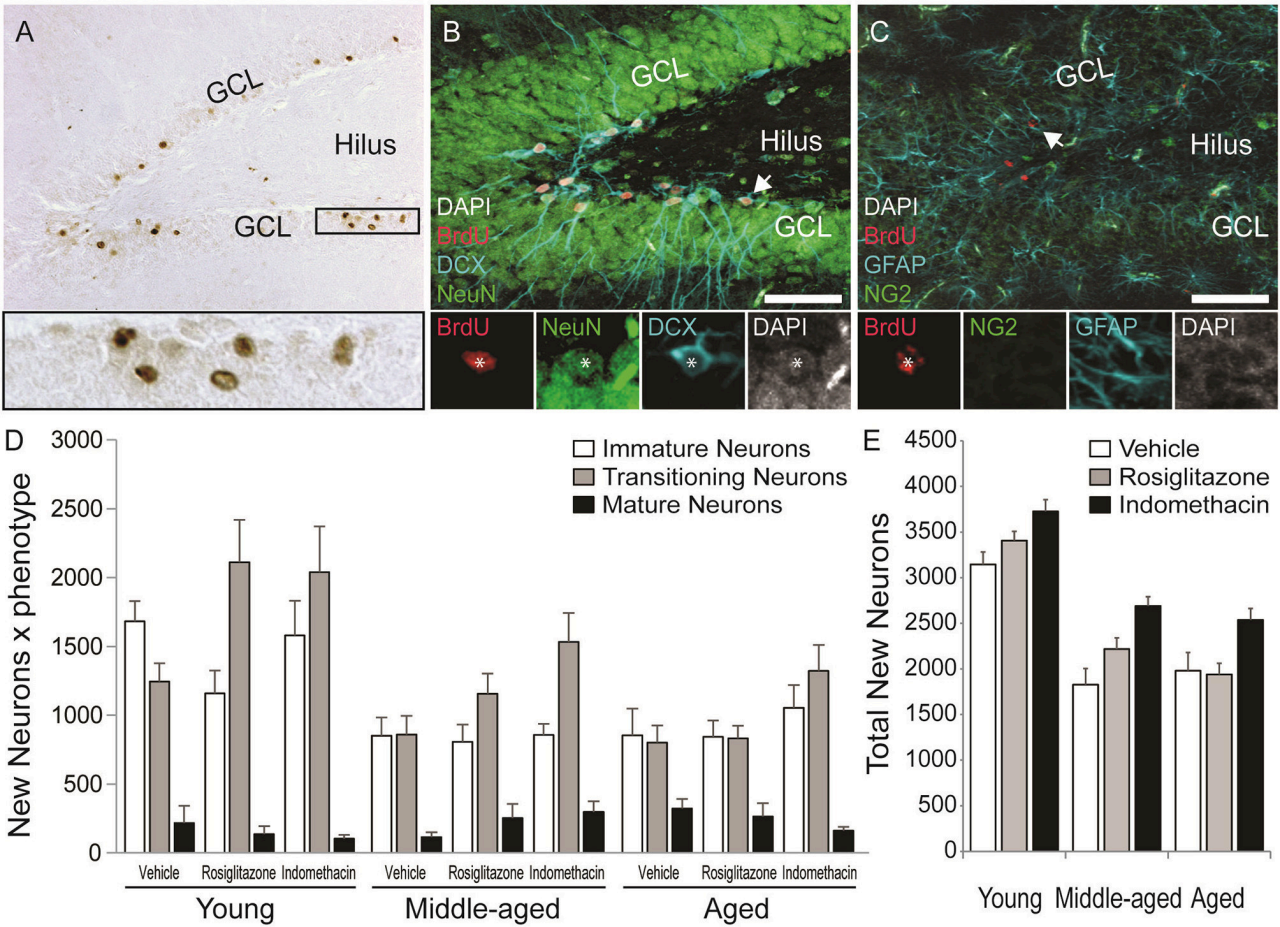
Age decreased but indomethacin treatment increased new neuron numbers. **(A)** Photomicrograph image of BrdU^+^ (in brown) cells taken under a 10x objective (inset shows BrdU^+^ cells under a 40x objective). Confocal images taken under a 20x objective with 1.5x digital zoom of **(B)** BrdU^+^ cells (in red) expressing immature DCX^+^ (in blue), transitioning (DCX^+^/NeuN^+^), and mature NeuN^+^ (in green) neuronal phenotypes and **(C)** Cells expressing NG2^+^ oligodendrocyte precursor and GFAP^+^ astrocyte phenotypes (in blue). The inset in **(B)** shows each marker for a transitioning neuron and in **(C)** a new astrocyte. **(D)** The mean (±S.E.M.) number of new immature (white bars), transitioning (gray bars) and mature (black bars) neurons in the hippocampi of vehicle-, rosiglitazone-, and indomethacin-treated young, middle-aged, and aged rats. Most new cells were transitioning (*p* < 0.0001 vs. other phenotypes) and then immature neurons (*p* < 0.0001 vs. mature neurons) and these more of these neurons were found in young vs. middle-aged and aged rats (all *p* < 0.0001). The number of transitioning neurons was highest (*p* < 0.01 vs. all other phenotypes) and new immature neurons next highest (*p* < 0.001 vs. mature neurons and glia) in young and middle-aged rats, but numbers of both phenotypes were similar in aged rats (both *p* < 0.0001 vs. mature neurons). Panel **(E)** shows the total number of new neurons in vehicle- (white bars), rosiglitazone- (gray bars), and indomethacin- (in black bars) treated rats. Young rats had more new neurons than middle-aged and aged (*p* < 0.001) rats and indomethacin-treated rats had more new neurons than vehicle- (*p* < 0.001) and rosiglitazone- (*p* < 0.01) treated rats.

Figure 5A shows representative examples of 10–12 d old BrdU^+^ cells revealed by DAB (in brown) in the dentate gyrus of a young vehicle-treated rat. Table 1 shows the total number of BrdU^+^ cells in the dentate gyri of young, middle-aged and aged rats in each drug treatment group. Total new cell numbers varied by age [*F*_(2, 38)_ = 28.71; *p* < 0.001] and treatment [*F*_(2, 38)_ = 9.39; *p* < 0.001] but not by the overall interaction [*F*_(4, 38)_ = 0.053; *p* = 0.99]. Young rats had more new cells than middle-aged (*p* < 0.001) and aged (*p* < 0.001) rats and indomethacin-treated rats had more new cells than vehicle- (*p* < 0.001) and rosiglitazone- (*p* < 0.05) treated rats.

**Table 1:** Hippocampal neurogenesis declines with age and is stimulated by indomethacin treatment.

**Age**	**Group**	**BrdU^+^ cell number**	**% of BrdU**^**+**^ **cells expressing phenotypic markers**					**Total new neurons and glia**					
			**Immature neuron**	**Transition neuron**	**Mature neuron**	**Astrocyte**	**OPCs**	**Immature neurons**	**Transition neurons**	**Mature neurons**	**Total neurons**	**Astrocytes**	**OPCs**
Y	Veh	3432.1 ± 209.8	49.7 ± 4.4	36.1 ± 2.8	5.8 ± 2.9	3.9 ± 0.8	1.4 ± 0.4	1683.0 ± 145.4	1243.7 ± 134.4	217.9 ± 124.9	3144.6 ± 211.4	129.1 ± 26.1	45.2 ± 10.8
	Rosi	3621.9 ± 346.5	33.3 ± 5.4	57.8 ± 3.9	3.3 ± 1.2	2.5 ± 0.7	1.6 ± 0.5	1159.4 ± 165.6	2113.1 ± 305.0	135.7 ± 60.0	3408.2 ± 295.8	98.9 ± 28.6	60.2 ± 18.6
	Indo	4019.7 ± 217.2	39.5 ± 5.7	50.3 ± 6.7	2.6 ± 0.6	4.7 ± 1.3	2.5 ± 0.3	1580.0 ± 252.7	2041.2 ± 332.0	104.9 ± 25.7	3726.1 ± 250.4	178.0 ± 40.7	96.7 ± 5.8
	Veh	2306.2 ± 94.8	36.3 ± 4.6	38.0 ± 6.7	5.3 ± 1.8	7.9 ± 1.3	5.1 ± 1.6	852.0 ± 131.5	858.5 ± 135.8	115.6 ± 36.4	1826.1 ± 112.2	186.2 ± 38.9	114.6 ± 34.7
MA	Rosi	2602.5 ± 236.7	30.4 ± 3.4	44.6 ± 4.0	9.0 ± 3.1	6.7 ± 0.7	6.7 ± 2.7	807.6 ± 124.3	1156.5 ± 147.1	252.2 ± 102.7	2216.3 ± 277.2	175.9 ± 27.9	153.0 ± 52.8
	Indo	3033.5 ± 136.9	28.7 ± 3.4	49.8 ± 4.8	10.2 ± 0.0	4.0 ± 1.4	5.2 ± 1.4	857.6 ± 79.1	1532.6 ± 210.8	298.4 ± 78.0	2688.6 ± 133.1	117.4 ± 42.3	163.7 ± 52.8
	Veh	2308.5 ± 91.3	36.7 ± 7.9	35.2 ± 5.9	13.8 ± 2.6	9.0 ± 2.8	4.9 ± 0.7	853.2 ± 194.4	801.9 ± 123.3	323.1 ± 67.4	1978.1 ± 082.4	201.3 ± 61.1	111.7 ± 15.2
A	Rosi	2651.5 ± 177.6	31.3 ± 3.0	32.4 ± 0.1	9.8 ± 3.1	11.2 ± 2.1	4.2 ± 1.4	843.0 ± 194.4	830.9 ± 123.3	263.5 ± 67.4	1937.4 ± 107.1	292.1 ± 55.2	112.1 ± 36.3
	Indo	3019.9 ± 101.2	35.4 ± 6.1	43.5 ± 5.4	5.4 ± 0.9	7.6 ± 2.1	2.7 ± 0.9	1053.1 ± 166.2	1322.2 ± 187.5	161.6 ± 27.1	2536.8 ± 075.7	230.6 ± 64.0	78.6 ± 25.8
Y		3671.7 ± 153.5	**41.4** ± **3.3^c^**	**47.3** ± **3.4^c^**	4.1 ± 1.1	3.7 ± 0.6	1.8 ± 0.2	**1487.2** ± **116.7^c^**	**1764.6** ± **173.5^d^**	156.9 ± 49.6	3408.7 ± 148.1	135.0 ± 18.9	66.0 ± 8.8
MA		**2626.1** ± **115.6^a^**	**32.0** ± **2.3^a^^,^^c^**	**43.8** ± **3.3^d^**	8.0 ± 1.5	6.3 ± 0.8	5.6 ± 1.1	**839.9** ± **63.3^a^^,^^c^**	**1162.3** ± **113.9^a^^,^^d^**	215.4 ± 44.4	**2217.6** ± **133.8^a^**	161.5 ± 21.5	142.0 ± 25.4
A		**2659.9** ± **103.9^a^**	**34.5** ± **3.3^a^^,^^c^**	**37.0** ± **3.2^a^^,^^c^**	9.7 ± 1.6	9.2 ± 1.3	4.0 ± 0.6	**916.4** ± **90.9^a^^,^^c^**	**985.0** ± **98.3^a^^,^^c^**	249.4 ± 41.3	**2150.8** ± **87.3^a^**	241.3 ± 33.7	100.8 ± 15.1
	Veh	2701.1 ± 155.7	**41.1** ± **3.4^c^**	**36.5** ± **2.9^c^**	8.0 ± 1.6	6.8 ± 1.1	3.7 ± 0.0	**1145.7** ± **129.9^c^**	**977.8** ± **87.1^c^**	212.7 ± 51.3	2336.1 ± 171.2	170.5 ± 24.0	89.3 ± 15.1
	Rosi	2958.6 ± 188.5	**31.7** ± **2.2**^b^^,^^c^	**45.0** ± **3.6**^b^^,^^d^	7.4 ± 1.6	6.8 ± 1.1	4.2 ± 1.1	**936.7** ± **85.1^c^**	**1366.9** ± **181.3**^b^^,^^d^	217.1 ± 49.7	2520.6 ± 214.0	189.0 ± 29.9	108.4 ± 23.0
	Indo	**3357.7** ± **151.4**^b^	**34.5** ± **3.0**^b^^,^^c^	**47.9** ± **3.1**^b^^,^^d^	6.1 ± 1.2	5.4 ± 1.0	3.4 ± 0.6	**1163.5** ± **126.3^c^**	**1632.0** ± **156.8**^b^^,^^d^	188.3 ± 34.4	**2983.9** ± **167.8**^b^	175.3 ± 29.5	113.0 ± 20.7

a *p < 0.05 vs. young*,

b *p < 0.05 vs. vehicle*,

c *p < 0.05 vs. mature neurons and glia within age or treatment*,

d *p < 0.05 vs. all other phenotypes within age or treatment. Significant differences are highlighted in bold*.

Figure 5 also shows representative examples of BrdU^+^ cells (in red) co-labeled with the (Figure 5B) the immature neuronal marker DCX (in blue) and/or the mature neuronal marker NeuN (in green) or (Figure 5C) the astrocyte marker GFAP (in blue) or the oligodendrocyte precursor marker NG2 (in green) in the dentate gyrus of a young vehicle-treated rat. Table 1 shows the mean (±S.E.M) % of BrdU^+^ cells expressing neuronal or glial phenotypes across age and treatment groups. These percentages varied by phenotype [*F*_(4, 152)_ = 204.31; *p* < 0.0001] and the interactions between age and treatment [*F*_(4, 38)_ = 2.94; *p* < 0.05], age and phenotype [*F*_(8, 152)_ = 3.55; *p* < 0.001] and phenotype and treatment [*F*_(8, 152)_ = 3.02; *p* < 0.01] but not by age, treatment, or other interactions between the independent variables (all *p* ≥ 0.14). Overall, the greatest % of BrdU^+^ cells expressed a transitioning (*p* < 0.0001 vs. all other phenotypes) followed by an immature (*p* < 0.0001 vs. mature neurons and glia) neuronal phenotype. However, the % of new cells expressing an immature neuronal phenotype was lower in middle-aged and aged vs. young rats (*p* < 0.01^a^ and *p* < 0.05^a^, respectively) and the % expressing a transitioning neuronal phenotype was lower in aged vs. young rats (*p* < 0.01^a^). In young and aged rats, the greatest % of new cells expressed an immature or transitioning neuronal phenotype (both *p* < 0.0001 vs. mature neurons and glia^c^) while in middle-aged rats the greatest % expressed a transitioning (*p* < 0.01 vs. all other phenotypes^d^) followed by an immature (*p* < 0.0001 vs. mature neurons and glia^*c*^) neuronal phenotype. Relative to vehicle, rosiglitazone and indomethacin increased the % of BrdU^+^ cells expressing a transitioning neuronal phenotype while decreasing the % expressing an immature neuronal phenotype (all *p* ≤ 0.05^b^). In vehicle-treated rats, the greatest percentages of BrdU^+^ cells expressed immature or transitioning neuronal phenotypes (both *p* < 0.001^c^) and in rosiglitazone and indomethacin-treated rats, greater percentages of cells expressed transitioning (both *p* < 0.001^d^ vs. all other phenotypes) followed by immature (both *p* < 0.001^c^ vs. mature neurons and glia) neuronal phenotypes.

Table 1 shows net neurogenesis or total mean (±S.E.M.) numbers of new neurons and glia and Figures 5D,E show the neuronal data separately. The number of new differentiated cells varied by age [*F*_(2, 38)_ = 35.02; *p* < 0.0001], treatment [*F*_(2, 38)_ = 10.57; *p* < 0.001] and phenotype [*F*_(4, 152)_ = 179.80; *p* < 0.0001] and numbers of neurons and glia varied by age [*F*_(8, 152)_ = 10.26; *p* < 0.0001] and by treatment [*F*_(8, 152)_ = 4.75; *p* < 0.0001] but not by other interactions between the independent variables (all *p* ≥ 0.23). Generally, more new differentiated cells were found in young vs. middle-aged (*p* < 0.0001) and aged (*p* < 0.0001) rats and in indomethacin-treated vs. vehicle- (*p* < 0.001) and rosiglitazone- (*p* < 0.01) treated rats. Most new cells were transitioning (*p* < 0.0001 vs. other phenotypes) followed by immature (*p* < 0.0001 vs. mature neurons and glia) neurons. Young rats had more new immature and transitioning neurons than middle-aged and aged rats (all *p* < 0.0001). In young and middle-aged rats, most new cells were transitioning neurons (*p* < 0.01 vs. all other cell types) followed by immature neurons (*p* < 0.001 vs. mature neurons and glia) and in aged rats, most new cells were immature or transitioning neurons (*p* < 0.0001 vs. mature neurons and glia). Table 1 and Figure 5E show that total new neuron numbers varied by age [*F*_(2, 38)_ = 42.52; *p* < 0.0001] and treatment [*F*_(2, 38)_ = 9.81; *p* < 0.001] but not by the overall interaction [*F*_(4, 38)_ = 0.46; *p* = 0.77]. Young rats had more new neurons than middle-aged (*p* < 0.001) and aged (*p* < 0.001) rats and indomethacin-treated rats had more new neurons than vehicle- (*p* < 0.001) and rosiglitazone- (*p* < 0.01) treated rats.

Dentate gyrus volumes (±S.E.M.) were calculated for young vehicle- (1.83 ± 0.03 mm^3^), indomethacin- (1.76 ± 0.02 mm^3^), and rosiglitazone- (1.73 ± 0.02 mm^3^) treated rats, middle-aged vehicle- (1.89 ± 0.03 mm^3^), indomethacin- (1.88 ± 0.07 mm^3^) and rosiglitazone- (1.89 ± 0.03 mm^3^) treated rats and aged vehicle- (1.86 ± 0.04 mm^3^), indomethacin- (1.88 ± 0.05 mm^3^) and rosiglitazone- (1.86 ± 0.03 mm^3^) treated rats. Age [*F*_(2, 38)_ = 6.60; *p* < 0.01] but not treatment [*F*_(2, 38)_ = 0.62; *p* = 0.54] or the overall interaction [*F*_(4, 38)_ = 0.07; *p* = 0.60] affected volumes. Young rats had smaller dentate gyri than middle-aged and aged rats (*p* < 0.01).

### Indomethacin and rosiglitazone modulate RMS neuroblast and OB_GCL_ new neuron densities

**Figure 6:**
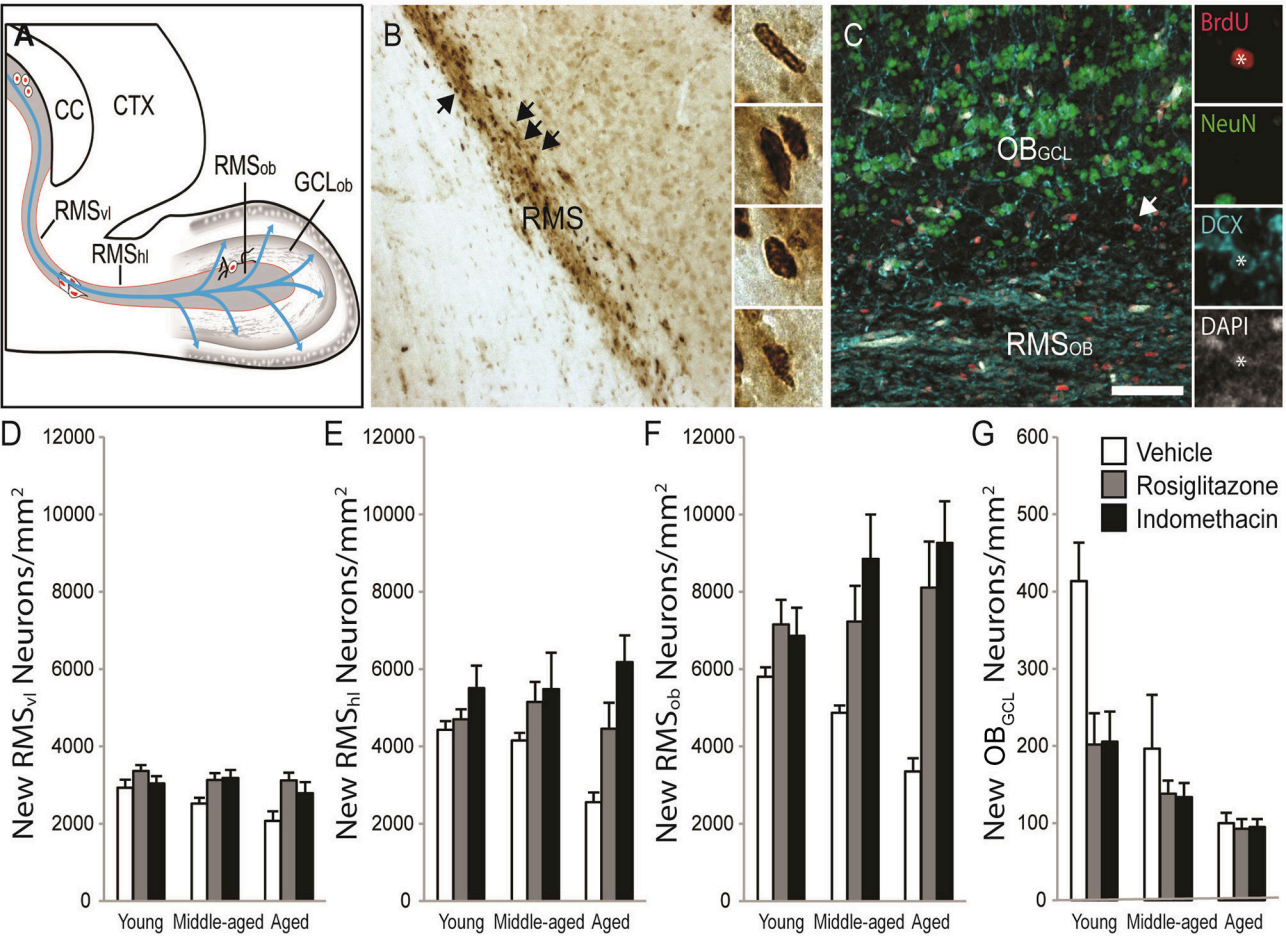
Indomethacin and rosiglitazone increase subependymal neurogenesis region-dependently. **(A)** A horizontal rat brain schematic showing the RMS_VL_, RMS_HL_, RMS_OB_, and OB_GCL_ where new cell densities and phenotypes were quantified. **(B)** Transmitted light photomicrograph of new BrdU^+^ cells revealed by DAB (in brown) migrating along the RMS_HL_ under a 20x objective. Arrows show examples of BrdU^+^ cells in the insets imaged under a 40x objective. **(C)** Confocal image of the olfactory bulb taken with a 20x objective (1.5 digital zoom). Most BrdU^+^ cells (in red) in the RMS expressed DCX (in blue) and a few BrdU^+^ cells in the OB_GCL_ expressed DCX and NeuN (in green). The inset shows each marker independently in a migrating BrdU^+^/DCX^+^ RMS_OB_neuroblast. The scale bar = 50 μm. **(D)** Densities of migrating neuroblasts were higher in the RMS_VL_ of young (*p* < 0.05) and tended to higher in the RMS_VL_ of middle-aged (*p* = 0.07) vs. aged rats and indomethacin (*p* < 0.05) and rosiglitazone (*p* < 0.01) treatment increased densities relative to vehicle. **(E)** In the RMS_HL_, densities of migrating neuroblasts were higher in indomethacin- vs. rosiglitazone- (*p* < 0.05) and vehicle- (*p* < 0.001) treated rats and in rosiglitazone- vs. vehicle- (*p* < 0.05) treated rats. **(F)** In the RMS_OB_, indomethacin (*p* < 0.001) and rosiglitazone (*p* < 0.001) increased neuroblasts/neuron densities relative to vehicle primarily because indomethacin increased densities in middle-aged rats (*p* <0.05 vs. vehicle) and indomethacin and rosiglitazone increased densities in aged rats (*p* <0.05 and *p* < 0.001 vs. vehicle, respectively). **(G)** Generally, young rats had higher OB_GCL_ neuron densities than middle-aged (*p* < 0.001) and aged (*p* < 0.001) rats and middle-aged rats tended to have higher densities than aged rats (*p* = 0.07). Indomethacin and rosiglitazone reduced densities relative to vehicle (*p* < 0.01), primarily because lower densities were observed in indomethacin- (*p* < 0.001) and rosiglitazone- (*p* < 0.05) vs. vehicle-treated young rats.

New RMS neuroblasts and OB neuron densities were quantified to test whether age-related declines occurred concomitantly with hippocampal neurogenesis and whether the declines could be reversed by drug treatment. The schematic in Figure 6A shows that neuroblasts chain migrate through the RMS_VL_, RMS_HL_ and then RMS_OB_ before migrating radially into OB_GCL_. Figure 6B shows representative examples of DAB-stained BrdU^+^ cells (in brown) in the RMS_HL_ and Figure 6C shows representative examples of BrdU^+^ cells (in red) expressing the immature neuronal protein DCX (in blue) and/or the mature neuronal protein NeuN (in green) in the RMS_OB_ and OB_GCL_. Note that BrdU^+^ cells expressing a mature DCX^−^/NeuN^+^ neuronal phenotype were not detected in the RMS or OB_GCL_ at the time point examined. The % of BrdU^+^/DCX^+^ was consistent across age [*F*_(2, 28)_ = 2.4; *p* = 0.11] and drug treatment [*F*_(2, 28)_ = 0.5; *p* = 0.63] and although a significant interaction was detected [*F*_(4, 28)_ = 4.1; *p* = 0.01], a Newman-Keuls post hoc test revealed that the % only tended to be higher in indomethacin- vs. vehicle-treated middle-aged rats (*p* = 0.10). Therefore, we simply report analyses of BrdU^+^/DCX^+^ densities in subependymal regions to avoid redundancy because > 95% of BrdU^+^ cells expressed DCX^+^ neuroblast or neuronal phenotypes and because BrdU^+^ cell densities correlated with BrdU^+^/DCX^+^ neuronal densities in the RMS_vl_[*r*_(37)_ = 0.998; *p* < 0.0001], RMS_hl_[*r*_(37)_ = 0.998; *p* < 0.0001], RMS_ob_ [*r*_(37)_ = 0.998; *p* < 0.0001], GCL_ob_ [*r*_(37)_ = 0.998; *p* < 0.0001] and hippocampal dentate gyrus [*r*_(37)_ = 0.920; *p* < 0.0001].

Table 2 and Figure 6D shows that migrating neuroblast densities in the RMS_VL_ varied by age [*F*_(2, 28)_ = 3.51; *p* < 0.04] and drug treatment [*F*_(2, 28)_ = 9.24; *p* < 0.001] but not by the overall interaction [*F*_(4, 28)_ = 1.02; *p* = 0.41]. Densities were higher in the RMS_VL_ of young (*p* < 0.05) and tended to higher in the RMS_VL_ of middle-aged (*p* = 0.07) vs. aged rats and were higher in the RMS_VL_ of indomethacin- (*p* < 0.05) and rosiglitazone- (*p* < 0.01) treated vs. vehicle-treated rats.

**Table 2:** Indomethacin and rosiglitazone increase subependymal neurogenesis.

	**Group**	**% DCX^+^(RMS/OB)**	**Neuroblast/Neuron #/mm2**				
			**DG**	**RMS_VL_**	**RMS_HL_**	**RMS_OB_**	**OB_GCL_**
Y	Veh	97.6 ± 0.2	65.9 ± 3.1	2934.0 ± 206.9	4435.1 ± 218.0	5801.4 ± 239.9	413.6 ± 49.9
	Rosi	96.8 ± 0.2	83.2 ± 5.7	3360.8 ± 152.2	4705.5 ± 255.6	7157.4 ± 639.5	**201.8** ± **40.7^b^**
	Indo	95.9 ± 0.2	79.1 ± 8.0	3039.8 ± 194.8	5512.4 ± 580.4	6861.5 ± 726.6	**205.3** ± **39.5^b^**
	Veh	95.9 ± 0.9	35.8 ± 2.2	2520.0 ± 152.4	4158.4 ± 194.5	4867.8 ± 194.8	**196.6** ± **69.4^a^**
MA	Rosi	96.6 ± 0.6	54.5 ± 2.8	3129.3 ± 179.7	5148.2 ± 524.1	**7229.0** ± **920.2^b^**	138.1 ± 17.3
	Indo	97.9 ± 0.1	46.5 ± 6.3	3186.6 ± 202.1	5480.4 ± 941.3	8848.1 ± 1151.2	133.5 ± 18.5
	Veh	97.9 ± 0.2	40.7 ± 1.1	2071.0 ± 249.6	2554.8 ± 252.0	3350.3 ± 345.8	**100.1** ± **13.4^a^**
A	Rosi	97.1 ± 0.4	51.3 ± 1.3	3126.8 ± 196.1	4461.7 ± 668.4	**8111.5** ± **1191.4^b^**	92.6 ± 12.4
	Indo	97.7 ± 0.6	39.1 ± 2.3	2789.5 ± 286.2	6185 ± 688.7	**9267.0** ± **1075.3^b^**	94.9 ± 10.3
Y		96.9 ± 0.2	75.4 ± 3.6	3116.6 ± 111.5	4839.5 ± 222.6	6588.6 ± 337.1	278.4 ± 36.6
MA		96.7 ± 0.4	**44.9** ± **2.9^a^**	2940.5 ± 130.2	4901.3 ± 334.6	6846.7 ± 638.2	**156.4** ± **23.9^a^**
A		97.6 ± 0.3	**43.7** ± **1.7^a^**	**2620.2** ± **191.9^a^**	4394.9 ± 568.2	6800.3 ± 957.9	**96.2** ± **6.4^a^**
	Veh	97.9 ± 0.2	47.8 ± 3.6	2541.1 ± 150.6	3771.4 ± 264.2	4760.0 ± 324.8	250.4 ± 47.0
	Rosi	97.1 ± 0.4	**54.9** ± **5.7^b^**	**3127.7** ± **98.3^b^**	**4819.5** ± **259.1^b^**	**7405.1** ± **477.7^b^**	**152.1** ± **20.2^b^**
	Indo	97.2 ± 0.6	**63.0** ± **4.3^b^**	**2988.8** ± **133.8^b^**	**5748.3** ± **688.7^b^**	**8278.0** ± **611.1^b^**	**145.6** ± **20.7^b^**

a *p < 0.05 vs. young*,

b *p < 0.05 vs. vehicle. DG densities were obtained by dividing the total numbers reported in Table 1 by the area that cells were counted upon. Significant differences are highlighted in bold*.

Table 2 and Figure 6E shows that migrating neuroblasts densities in the RMS_HL_ varied by treatment [*F*_(2, 28)_ = 12.51; *p* < 0.001] but not by age [*F*_(2, 28)_ = 1.02; *p* = 0.38] or the overall interaction [*F*_(4, 28)_ = 2.25; *p* = 0.09]. Indomethacin increased densities relative to rosiglitazone (*p* < 0.05) and vehicle (*p* < 0.001) and rosiglitazone increased densities relative to vehicle (*p* < 0.05).

Table 2 and Figure 6F shows that migrating neuroblast/new neuron densities in the RMS_OB_ varied by treatment [*F*_(2, 28)_ = 19.25; *p* < 0.001] and the overall interaction [*F*_(4, 28)_ = 3.02; *p* < 0.05] but not by age [*F*_(2, 28)_ = 0.22; *p* = 0.80]. Specifically, indomethacin (*p* < 0.001) and rosiglitazone (*p* < 0.001) increased densities relative to vehicle primarily because indomethacin increased densities in middle-aged rats (*p* < 0.05 vs. vehicle) and indomethacin and rosiglitazone increased densities in aged rats (*p* < 0.05 and *p* < 0.001 vs. vehicle, respectively).

Table 2 and Figure 6G shows that new neuron densities in the OB_GCL_ varied by age [*F*_(2, 28)_ = 16.61; *p* < 0.001], treatment [*F*_(2, 28)_ = 5.74; *p* < 0.01] and the overall interaction [*F*_(4, 28)_ = 2.67; *p* ≤ 0.05]. Generally, young rats had higher densities than middle-aged (*p* < 0.001) and aged (*p* < 0.001) rats and middle-aged rats tended to have higher densities than aged rats (*p* = 0.07). Indomethacin and rosiglitazone reduced densities relative to vehicle (*p* < 0.01), primarily because lower densities were observed in indomethacin- (*p* < 0.001) and rosiglitazone- (*p* < 0.05) vs. vehicle-treated young rats.

Finally, new dentate gyrus neuron densities were affected by age [*F*_(2, 28)_ = 56.44; *p* < 0.0001] and drug treatment [*F*_(2, 28)_ = 10.44; *p* < 0.001] but not the overall interaction [*F*_(4, 28)_ = 0.91; *p* = 0.47]. Higher new neuron densities were found in the dentate gyri of young vs. middle-aged (*p* < 0.001) and aged (*p* < 0.001) rats and following treatment with indomethacin (*p* < 0.05 vs. rosiglitazone and *p* < 0.001 vs. vehicle) or rosiglitazone (*p* < 0.05 vs. vehicle; Table 2).

While new dentate gyrus neuron densities neither correlated with RMS_VL_, RMS_HL_, or RMS_OB_ neuroblast densities nor new OB_GCL_ neuron densities in young and middle-aged rats, new DG neuron densities tended to correlate positively with RMS_OB_ neuroblast densities [*r*_(11)_ = 0.58; *p* = 0.06] in aged rats. Interestingly, RMS_OB_ densities correlated negatively with immature OB_GCL_ neuron densities [*r*_(14)_ = −0.73; *p* < 0.01] in young rats, RMS_HL_ densities correlated positively with RMS_OB_ neuroblast densities [*r*_(12)_ = 0.88; *p* < 0.001] in middle-aged rats and RMS_OB_ neuroblast densities correlated positively with both RMS_VL_ [*r*_(11)_ = 0.71; *p* < 0.05] and RMS_HL_ [*r*_(11)_ = 0.70; *p* < 0.05] neuroblast densities in aged rats, suggesting that age impacts neuroblast migration.

### Rosiglitazone treatment increases proportions of resting microglia in aging rats

**Figure 7:**
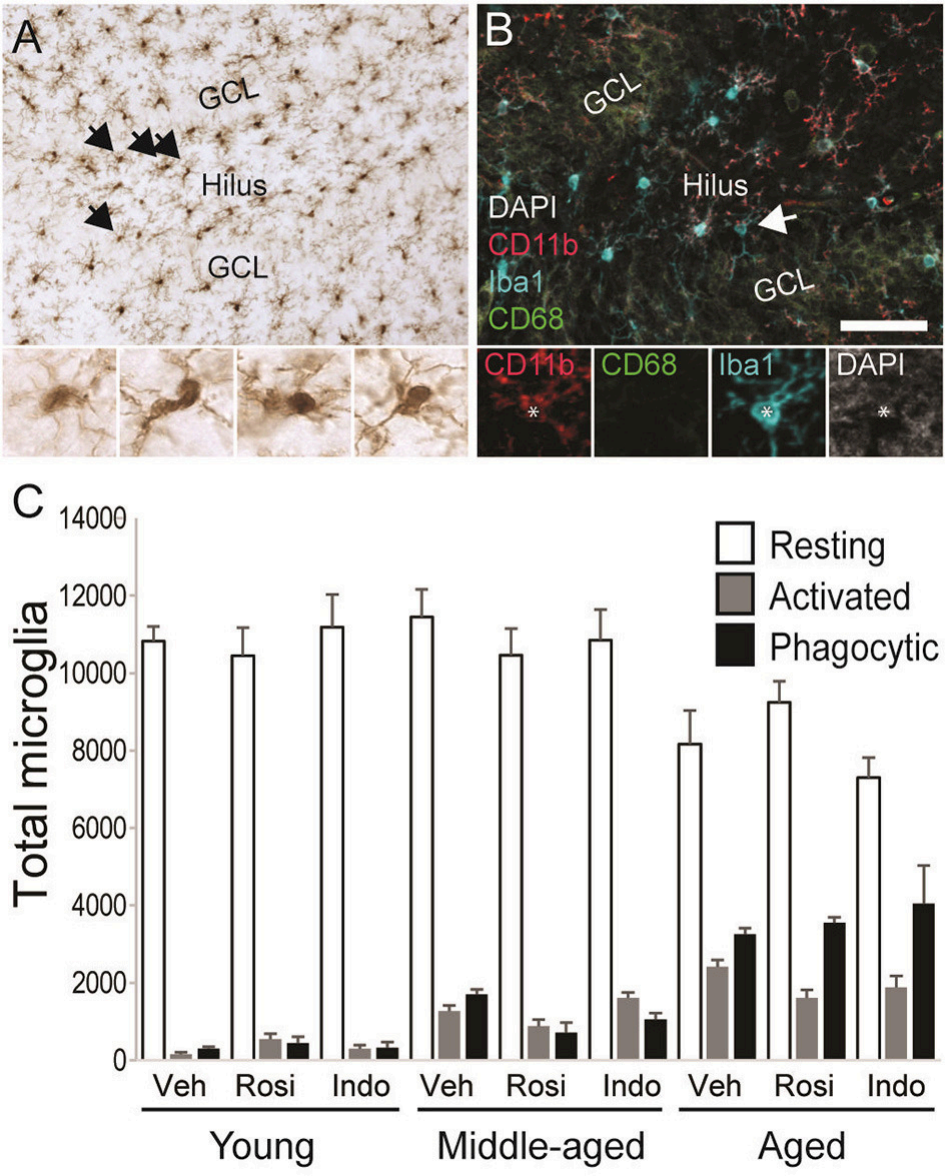
Microglial activation increased with age. **(A)** A representative transmitted light photomicrograph of the dentate gyrus showing Iba1^+^ microglia (in brown). The arrows indicate Iba1^+^ cells that are shown in the insets under 40x. **(B)** A confocal image of the dentate gyrus taken under a 20x objective (with 1.5x digital zoom) showing Iba1^+^ cells (in blue), the marker of activation CD11b (in red), the phagocyte marker CD68 (in green) and the nuclear stain DAPI (in gray). The total number of resting Iba1^+^ microglia, activated Iba1^+^/CD11b^+^ microglia and phagocytic Iba1^+^/CD11b^+^/CD68^+^ microglia were calculated. The arrow shows each marker independently in a phagocytic microglia. The scale bar = 50 μm. **(C)** shows the total number of resting (white bars), activated (gray bars), and phagocytic (black bars) microglia in vehicle-, rosiglitazone, and indomethacin-treated young, middle-aged, and aged rats. Generally, microglia numbers were higher in middle-aged and aged vs. young rats (both *p* < 0.01) and more resting (*p* < 0.001 vs. activated and phagocytic) followed by phagocytic (*p* < 0.05 vs. activated) microglia were detected. Resting microglia numbers were higher in young vs. aged rats (*p* < 0.001), activated microglia numbers were higher in aged vs. young rats (*p* < 0.001) and phagocytic microglia numbers higher in aged (*p* < 0.001 vs. middle-aged and young rats) followed by middle-aged (*p* < 0.05 vs. young rats) rats. In young and middle-aged rats, resting microglia outnumbered activated and phagocytic microglia (all *p* < 0.001). In aged rats, resting microglia outnumbered activated and phagocytic microglia (both *p* < 0.001) and phagocytic microglia outnumbered activated microglia (*p* < 0.001).

Figure 7A shows representative examples of DAB-stained Iba1^+^ microglia and Figure 7B shows representative examples of Iba1^+^ microglia (in blue) expressing the activation marker CD11b (in red) or the phagocytosis marker CD68 (in green) in the dentate gyrus of an aged vehicle-treated rat. Table 3 shows total Iba1^+^ microglia numbers, the percentages of Iba1^+^ microglia expressing CD11b^−^/CD68^−^ (resting), CD11b^+^/CD68^−^ (activated) and CD11b^+^/CD68^+^ (phagocytic) phenotypes and along with Figure 7C the total number of resting, activated and phagocytic microglia in the dentate gyri of young, middle-aged, and aged rats in each treatment group. The total number of Iba-1^+^ microglia varied by age [*F*_(2, 38)_ = 6.35; *p* < 0.01] but not by treatment [*F*_(2, 38)_ = 0.32; *p* = 0.73] or the overall interaction [*F*_(4, 38)_ = 1.16; *p* = 0.34]. More Iba1^+^ microglia were found in the dentate gyri of middle-aged (*p* < 0.01) and aged (*p* < 0.01) vs. young rats.

**Table 3:** Numbers of activated and phagocytic microglia increase with age.

**Age**	**Group**	**Iba1^+^ Cell #**	**% Iba1**^**+**^ **of Cells by phenotype**			**Total microglia number by phenotype**		
			**Resting**	**Activated**	**Phagocytic**	**Resting**	**Activated**	**Phagocytic**
Y	Veh	11265.0 ± 374.3	96.0 ± 0.4	2.0 ± 0.3	2.3 ± 0.6	10819.1 ± 384.1	153.1 ± 54.9	292.7 ± 59.2
	Rosi	11433.6 ± 641.7	91.2 ± 2.3	5.2 ± 1.2	3.8 ± 1.3	10449.5 ± 722.8	545.6 ± 144.3	438.4 ± 173.8
	Indo	11815.2 ± 835.8	94.6 ± 0.7	2.6 ± 0.8	2.9 ± 1.0	11190.6 ± 838.4	298.9 ± 90.1	325.6 ± 143.1
	Veh	14416.0 ± 650.9	79.1 ± 2.0	8.9 ± 1.1	11.9 ± 0.9	11441.0 ± 713.8	1272.7 ± 143.9	1702.1 ± 120.8
MA	Rosi	12052.8 ± 756.7	**86.7** ± **2.4^c^**	7.5 ± 1.7	5.8 ± 1.6	10460.8 ± 693.9	882.1 ± 165.6	709.80 ± 261.3
	Indo	13507.2 ± 528.6	80.0 ± 2.6	12.0 ± 1.3	8.0 ± 1.5	10856.9 ± 786.2	1601.1 ± 153.8	1049.0 ± 174.0
	Veh	13824.0 ± 1139.7	58.6 ± 1.7	17.4 ± 0.7	23.8 ± 1.3	8167.6 ± 870.2	2408.3 ± 189.2	3248.0 ± 156.7
A	Rosi	14397.6 ± 531.1	**64.0** ± **1.9^c^**	11.2 ± 1.3	24.8 ± 1.4	9240.5 ± 550.3	1608.1 ± 207.8	3548.8 ± 151.3
	Indo	13238.4 ± 1599.5	56.9 ± 4.2	13.98 ± 0.6	29.1 ± 3.7	7304.9 ± 515.5	1883.0 ± 291.4	4050.4 ± 974.8
Y		11489.6 ± 337.0	**94.0** ± **0.8^e^**	3.1 ± 0.5	2.9 ± 0.5	**10819.7** ± **355.3^e^**	321.3 ± 67.4	348.5 ± 70.1
MA		**13393.5** ± **433.3^a^**	**81.7** ± **1.5^a^^,^^e^**	**9.4** ± **0.8^a^**	**8.8** ± **0.9^a^**	**10952.2** ± **408.0^e^**	**1253.3** ± **110.6^f^**	**1187.9** ± **147.6^a^**
A		**13820** ± **640.5^a^**	**59.8** ± **1.7^a^^,^^b^**	**14.2** ± **0.8^a^^,^^b^**	**25.9** ± **1.4^a^^,^^b^^,^^d^**	**8237.67** ± **413.6^a^^,^^b^^,^^e^**	**1966.5** ± **153.2^a^**	**3615.7** ± **320.7^a^^,^^b^^,^^d^**
	Veh	13129.7 ± 528.9	79.0 ± 3.8	9.0 ± 1.6	12.0 ± 2.2	10258.7 ± 496.3	1211.6 ± 237.4	1659.3 ± 302.3
	Rosi	12628 ± 486.8	80.6 ± 3.4	8.0 ± 1.0	11.4 ± 2.6	10050.3 ± 384.5	1011.9 ± 150.8	1565.7 ± 391.0
	Indo	12853.6 ± 613.3	77.1 ± 4.4	9.5 ± 1.4	13.3 ± 3.2	9784.16 ± 609.9	1261.0 ± 212.4	1808.3 ± 530.1

a *p < 0.05 vs. young*,

b *p < 0.05 vs. middle-aged*,

c *p < 0.05 vs. vehicle within age or treatment*,

d *p < 0.05 vs. activated within age or treatment*,

e *p < 0.05 vs. all other phenotypes within age or treatment*,

f *p = 0.06 vs. young. Significant differences are highlighted in bold*.

The % of Iba1^+^ cells expressing activation markers varied by phenotype [*F*_(2, 76)_ = 3026.47; *p* < 0.0001] and the interactions between phenotype and age [*F*_(4, 76)_ = 151.61; *p* < 0.0001] and phenotype, age and drug treatment [overall interaction: *F*_(8, 76)_ = 3.19; *p* < 0.01] but was not affected by age, drug treatment or other interactions between the independent variables (all *p* ≥ 0.09). The greatest % of Iba1^+^ microglia exhibited a resting (*p* < 0.001 vs. phagocytic and activated) followed by a phagocytic (*p* < 0.001 vs. activated) phenotype. The % of resting microglia was higher while the percentages of activated and phagocytic microglia were lower in young vs. middle-aged rats and aged rats and in middle-aged vs. aged rats (all *p* < 0.001). These percentages were unaffected by drug treatment in young rats but rosiglitazone increased the % of resting microglia in middle-aged rats relative to vehicle (*p* < 0.01) and in aged rats relative to vehicle (*p* < 0.05) and indomethacin (*p* < 0.05).

These effects translated into group differences in total resting, activated and phagocytic microglia numbers. Microglial numbers varied by age [*F*_(2, 38)_ = 6.25; *p* < 0.01], phenotype [*F*_(2, 76)_ = 1148.66; *p* < 0.0001] and the interaction between phenotype and age [*F*_(4, 76)_ = 41.41; *p* < 0.0001] but not by treatment or other interactions between the dependent variables (all *p* ≥ 0.12). Overall, more resting microglia were detected than activated (*p* < 0.001) or phagocytic (*p* < 0.001) microglia and more phagocytic microglia were detected than activated microglia (*p* < 0.05). The latter effect was largely due to the increase phagocytic microglia numbers detected in aged rats (young rats: resting microglia > activated and phagocytic microglia; *p* < 0.001; middle-aged rats: resting microglia > activated and phagocytic microglia; *p* < 0.001; aged rats: resting microglia > phagocytic microglia > activated microglia; *all p* < 0.001). In fact, fewer resting microglia were detected in aged vs. young (*p* < 0.001) and middle-aged rats (*p* < 0.001), more activated microglia were detected in aged vs. young rats (*p* < 0.001), and the number of phagocytic microglia was highest in aged rats (vs. middle-aged and young rats; *p* < 0.001) followed by middle-aged rats (vs. young rats; *p* < 0.02).

### Relationships between neurogenesis, microglia, and behavior

Table 4 shows correlations between new neurons, microglia, and behavioral measures obtained from the 2nd water maze session within each age group. In young rats, new neuron numbers correlated positively with immediate probe DI scores [*r*_(16)_ = 0.56; *p* < 0.05] and the % time spent in the goal quadrant [*r*_(16)_ = 0.50; *p* ≤ 0.05]. Total activated microglia numbers correlated positively with DI scores [*r*_(16)_ = 0.62; *p* < 0.01] and the % time spent in the target quadrant [*r*_(16)_ = 0.54; *p* < 0.05] on the delayed probe trial and with the difference in DI scores obtained from the delayed probe trial in the 2nd vs. 1st water maze session [*r*_(16)_ = 0.51; *p* < 0.05]. Phagocytic microglia numbers correlated negatively with the % time spent in the target quadrant on the immediate probe trial [*r*_(16)_ = −0.65; *p* < 0.01] and delayed probe trial [*r*_(16)_ = −0.51; *p* < 0.01]. In middle-aged rats, new neuron number correlated negatively with average pathlength across hidden trial blocks [*r*_(15)_ = −0.78; *p* < 0.001] and phagocytic microglia number [*r*_(15)_ = −0.52; *p* < 0.05]. In aged rats, resting microglia numbers correlated negatively with DI scores [*r*_(15)_ = −0.64; *p* < 0.01] and % time spent in the target quadrant [*r*_(15)_ = −0.77; *p* < 0.001] on the immediate probe trial and resting microglia numbers correlated negatively with the difference in % time spent in the target quadrant on the delayed probe trial in the 2nd vs. 1st session [*r*_(15)_ = −0.56; *p* < 0.05].

**Table 4:** Correlations between new neurons, microglia, and behavioral scores on the 2nd session.

**Age**	**Total cell #**			**Immediate probe**		**Delayed probe**		**2nd vs. 1st Memory Session Diff Scores**	
		**New Neurons**	**Mean PL**	**DI score**	**% Time TQ**	**DI score**	**% Time**	**Δ DI**	**Δ %*t***
Y	New neurons microglia	−	0.04	**0.56^a^**	0.49	−0.06	−0.27	0.02	−**0.58^a^**
	Resting	0.19	0.13	−0.05	−0.16	−0.25	−0.38	−0.43	−0.26
	Activated	−0.13	−0.39	−0.23	0.01	**0.62^b^**	**0.53^a^**	**0.51^a^**	0.50
	Phagocytic	−0.23	0.04	−0.48	−**0.65^b^**	−**0.51^a^**	−0.45	−0.11	0.00
MA	New neurons microglia	−	−**0.78^c^**	0.04	0.05	0.28	0.15	0.24	0.05
	Resting	−0.37	0.25	−0.03	0.06	−0.04	0.00	−0.04	−0.06
	Activated	0.06	0.09	−0.07	−0.11	0.03	−0.09	−0.03	0.05
	Phagocytic	−**0.52^a^**	0.46	0.13	0.05	0.04	0.00	−0.01	−0.05
A	New neurons microglia	−	−0.39	0.21	0.24	−0.05	−0.20	−0.08	−0.37
	Resting	−0.32	0.24	−**0.64^b^**	−**0.77^c^**	−0.33	−0.19	−0.31	**0.56^a^**
	Activated	−0.32	0.16	−0.49	−0.25	−0.45	−0.22	−0.33	0.06
	Phagocytic	0.09	−0.14	−0.19	−0.23	−0.25	−0.09	−0.14	0.14

a *p < 0.05*,

b *p < 0.01*,

c *p < 0.001*.

*Significant relationships are highlighted in bold*.

## Discussion

In this study, we tested whether the broad spectrum NSAID indomethacin and PPARγ activator rosiglitazone could rejuvenate cognition and neurogenesis. In the first water maze session, young rats outperformed aged rats but all rats learned information about the hidden platform location after abbreviated training. However, only about one half of all rats remembered the platform location after a 24 h delay. We capitalized upon this performance variability to assign rats uniformly to treatment groups based upon age and combined probe trial performances. In the second water maze session, all rats learned a novel hidden platform location after abbreviated training and middle-aged and aged rats actually outperformed young rats on the probe trial administered 24 h later. Difference scores showed that indomethacin potentiated the improvement in delayed probe trial performance exhibited by middle-aged rats. Middle-aged rats with more new hippocampal neurons exhibited shorter average pathlengths across training trials in the 2nd water maze session and had fewer phagocytic microglia. Indomethacin increased new hippocampal neurons across age groups and both drugs differentially increased neurogenesis across subependymal regions. New hippocampal granule neuron numbers tended to correlate with RMS_OB_ neuroblasts densities, but only in aged rats, suggesting that the mechanisms mediating age-related declines in neurogenesis are region-dependent. Overall, our data suggest the feasibility of testing longer-term immunomodulatory strategies for treating age-related declines in spatial ability and neurogenesis.

Although, expected age-related spatial impairments were observed in the 1st water maze session (Foster et al., 2003; Foster and Kumar, 2007; Carter et al., 2009; Speisman et al., 2013a,b; Scheinert et al., 2015), all age groups acquired information about the hidden platform location. We confirmed better performances on final vs. initial training block bins across age groups and that all age groups discriminated the target quadrant on the immediate probe trial (Figures 2C,D). However, all age groups performed more poorly on the 24 h memory probe trial (Figures 2E,F) than similar age groups in our previous studies that employed an additional training trial block before and a refresher training trial block after the immediate probe trial. These trial blocks were eliminated from the current study to minimize the probability that memory of the platform location learned in the 1st water maze session would interfere with the rapid acquisition of the novel platform position in the 2nd water maze session (Gerlai, 2001; Ormerod and Beninger, 2002; Speisman et al., 2013b). While we capitalized on combined probe trial variability to assign rats in each age group uniformly to treatment groups in the current study and to identify improved intersession delayed probe trial performances in middle-aged rats, future experiments employing repeated measures water maze protocols should likely optimize training trial blocks to promote better delayed memory probe performances in young rats.

In the 2nd water maze session, all rats learned a novel hidden platform location but aging rats surprisingly outperformed young rats on the memory probe. Young rats typically outperform aged rats on delayed water maze probe trials after massed or distributed training sessions (Wyss et al., 2000; Driscoll et al., 2006; Speisman et al., 2013b) and outperformed aging rats on hidden platform training trials before performing similarly and then worse on respective immediate and delayed probe trials in the 2nd water maze session of the current study. We could have revealed the well-documented decline in age-related cognitive flexibility (Barense et al., 2002; Schoenbaum et al., 2002; Nicolle and Baxter, 2003; Bizon et al., 2012; Breton et al., 2015; Pereira et al., 2015) by increasing ambiguity about the rules for solving the task by reducing the number of training trial blocks. In addition, the increased water maze temperature that we employ to minimize hypothermia and temperature-induced memory impairments in aging rats (Lindner and Gribkoff, 1991; Salehi et al., 2010) may have insufficiently motivated young rats to retain information about the hidden platform location over the 24 h delay. The slightly higher (albeit clinical) drug treatment dosages employed in young vs. aging rats could have also impaired memory. Future experiments testing the effects of these variables may shed light upon why undertrained young rats performed just above chance levels on the delayed probe trial in the 2nd water maze session. Nonetheless, we add to our previous caution that the *rapid* water maze task may not identify reliable or valid relationships between neurogenesis and spatial ability in young rats (Speisman et al., 2013b) by showing that young undertrained rats exhibited unexpected delayed probe trial performances in this task.

We found that indomethacin potentiated the improvements in delayed probe trial performances exhibited by middle-aged rats on the 2nd vs. 1st water maze session (Figure 4). Indomethacin broadly inhibits COX-1 and COX-2 activity that catalyzes prostaglandin, prostacyclin and thromboxane activity and also activates central PPAR-γ proteins that transcriptionally inhibit COX-2 and numerous pro-inflammatory cytokines (Jiang et al., 1998; Dannhardt and Kiefer, 2001; Monje et al., 2003). Indomethacin has been shown to protect spatial cognition from inflammation in young rodents and improve cognition in a scopolamine model of aging (Monje et al., 2003; Kanno et al., 2012; Ormerod et al., 2013) but to our knowledge, this is the first demonstration that oral indomethacin improves water maze performance in a natural rodent aging model. More selective PPAR-γ activation has been shown to improve spatial/contextual cognition in middle-aged rats (Gemma et al., 2004; Wang et al., 2012) but our shorter rosiglitazone course (7 d vs. 40–60d) did not improve memory in middle-aged or aged rats. A longer or higher-dose course or the more specific PPAR-γ activator rosiglitazone may be required to produce the same cognitive benefit for aging rats that the same short course of broad spectrum indomethacin produced in middle-aged rats. In addition, a longer indomethacin course may be required to produce the same cognitive benefit for aged rats that we observed in middle-aged rats that exhibited milder signs of neuroinflammation (Table 3). These observations support the feasibility of testing the effects of longer-term NSAID treatments with variable specificities on age-related cognitive decline.

Our data and mixed clinical findings support the hypothesis that the efficacy of NSAID treatment for age-related cognitive decline will likely reflect interactions between specificity, treatment duration, disease etiology, stage of progression and the number and type of evaluations employed (Waldstein et al., 2010). For example, neither selective nor broad spectrum NSAID treatment appears to improve Alzheimer's patient outcomes when initiated after the symptoms become clinically obvious (Aisen et al., 2000, 2003; Van Gool et al., 2001; Martin et al., 2008) and likely reflect significant neuronal loss. Long-term low-dose aspirin treatment for vascular disease prophylaxis has been reported to protect (Anthony et al., 2000; Zandi et al., 2002; Nilsson et al., 2003), provide no benefit (Henderson et al., 1997; Stewart et al., 1997; Kang et al., 2007) and even increase Alzheimer's and vascular dementia risk (Hébert et al., 2000; Cornelius et al., 2004). However when dementia, hypertension and cardiovascular disease are controlled for, long-term aspirin and NSAID users respectively exhibit better longitudinal learning and memory scores and less prospective cognitive decline on tests of memory, concentration, and mental flexibility than controls (Rozzini et al., 1996; Stürmer et al., 1996; Hayden et al., 2007; Waldstein et al., 2010). Although, specific COX-2 inhibition has been reported to impair memory potentially by compromising long-term potentiation and neurogenesis in rodent models (Sharifzadeh et al., 2005a,b; Cowley et al., 2008; Goncalves et al., 2010; Ormerod et al., 2013), a randomized double-blind study showed that celecoxib-treated subjects initially reporting memory complaints exhibited higher memory test scores and elevated prefrontal cortical metabolic and activity levels than placebo-treated controls (Small et al., 2008). Combined, these data suggest that testing the safety and efficacy of immunomodulatory strategies is feasible for age-related cognitive decline that precedes or escapes dementia.

We observed the expected age-related decrease in hippocampal neurogenesis and found that indomethacin increased new hippocampal neuron number, regardless of age (Monje et al., 2003; Ormerod et al., 2013). As expected, most 10–12 d old cells expressed a transitioning followed by an immature neuronal phenotype (Brandt et al., 2003; Kempermann et al., 2004; Keene et al., 2009; Breunig and Rakic, 2010; Lugert et al., 2010; Ormerod et al., 2013; Speisman et al., 2013a,b) and both of these phenotypes were reduced in the hippocampi of aging rats. Although, we did not specifically test the effects of age or drug treatment on stages of neurogenesis, our results are consistent with those showing that age primarily compromises NPC proliferation and perhaps neuronal differentiation and survival (Cameron and McKay, 1999; Rao et al., 2006; Olariu et al., 2007; Lazarov et al., 2010; Marlatt et al., 2012). While we found that both indomethacin and rosiglitazone increased the number of transition-state neurons, only the effect of indomethacin was robust enough to increase total new neuron numbers. Young neurons are particularly vulnerable to death at about 2 weeks after birth (Cameron et al., 1993; Dayer et al., 2003), which may explain the beneficial effects of indomethacin and rosiglitazone treatment on neurogenesis in the hippocampi of young rats. While drug treatment could have modulated a low-level immune response in these rats that were housed in standard colony conditions, it could have also modulated an as yet unidentified local response that renders these 10–12 d old neurons vulnerable to death. Future experiments specifically testing the effects of NSAID treatment on stages of neurogenesis could provide insight about how to rejuvenate neurogenesis later in life.

Age-related decreases in hippocampal neurogenesis could underlie age-related cognitive decline. In young rats, new neuron number correlated positively with immediate probe DI score but negatively with the change in % time spent in the training quadrant on the 2nd vs. 1st session. If neurogenesis supports hippocampal integrity, then these data support the notion that undertrained young rats readily learn a hidden platform location but changed strategies on the memory probe trial in this abbreviated task. Middle-aged and aged rats with more new neurons swam more directly to the novel hidden platform location in the 2nd water maze session, but the positive correlation between these variables only achieved statistical significance for the middle-aged group. In fact, the survival time of ~2.5 weeks after the onset of drug treatment and 10–12 d after the final BrdU injection was likely too short to test the strength of the relationships between drug-produced new neurons and water maze scores in aging rats, since new neurons only fully mature after a month or more (Cameron and McKay, 2001; van Praag et al., 2002; Ormerod et al., 2013). In addition, longer exposures to other interventions, such as daily exercise or enriched environments that increase neurogenesis can also improve scores in spatial tasks (Drapeau et al., 2003, 2007; van Praag et al., 2005; Dupret et al., 2008; Speisman et al., 2013a,b; Scheinert et al., 2015). In fact, studies that have specifically manipulated BrdU injections and survival times report that neurogenesis rates can predict spatial task scores and that appropriately timed spatial training can potentiate the survival of new neurons (Gould et al., 1999a; Drapeau et al., 2003, 2007; Epp et al., 2007, 2010). Future work employing longer treatment protocols and survival times between BrdU injections and water maze retraining and testing would likely reveal stronger relationships between indomethacin- and rosiglitazone-induced increases in new neuron numbers and behavioral scores in aging rats.

A sub-goal of the study was to test whether neurogenesis declines with age in a region-dependent or global manner to identify potential mechanisms of the decline. Therefore, we tested the strength of relationships between RMS neuroblast, new OB neuron and new hippocampal granule neuron densities in individual rats. Consistent with other studies showing that new cells migrate from the rodent subventricular zone (SVZ) to the olfactory bulbs in about 10–14 days (Gritti et al., 2002; Carleton et al., 2003; Whitman and Greer, 2007), new RMS and OB BrdU^+^ cells expressed an immature DCX^+^ neuroblast/neuronal phenotype or a transitioning DCX^+^/NeuN^+^ neuronal phenotype but not a mature DCX^−^/NeuN^+^ phenotype. Relative to young rats, new OB_GCL_ neuron densities were lower in middle-aged and aged rats and RMS_VL_ neuroblast densities were lower in aged rats. These data are consistent with published work showing that SVZ NPC proliferation declines with age, but also suggest that the survival of new OB interneurons may become compromised in middle age and the proliferation of migrating RMS neuroblasts may become compromised with more advanced age (Tropepe et al., 1997; Enwere et al., 2004; Capilla-Gonzalez et al., 2013; Mobley et al., 2013). Although, previous work did not detect effects of age on RMS neuroblast migration (Mobley et al., 2013), the correlated densities of new cells in subependymal regions (i.e., anterior RMS in young rats, medial RMS in middle-aged rats and more distributed across the RMS in aged rats) suggest that future work should specifically test the effect of age on RMS neuroblast migration. RMS_OB_ densities tended to correlate with hippocampal granule neuron densities, but only in aged rats. Coupled with our finding that new OB neuron densities begin to decline in middle age while RMS_VL_ densities decline later in life, this relationship tempts speculation that region-specific mechanisms may mediate declining neurogenesis in middle age that can become aggravated and perhaps coordinated by systemic mechanisms later in life.

The expression of many factors that regulate adult mammalian neurogenesis has been shown to change with age. Neurogenesis can be rejuvenated by growth factors and by exposure to exercise or enrichment that stimulate growth factor production in the aged brain (Tropepe et al., 1997; Lee et al., 2002; Aberg et al., 2003; Shetty et al., 2005; David Aberg et al., 2010). Downregulated mechanistic target of rapamycin (mTOR) and endocannabinoid signaling may play a role in reduced neuron production across lifespan (Goncalves et al., 2008; Paliouras et al., 2012). The phytoalexin resveratrol and the leukotriene inhibitor and anti-asthmatic drug montelukast both modulate neuroinflammation, stimulate angiogenesis, increase neurogenesis and improve learning and memory in aged rats (Kodali et al., 2015; Marschallinger et al., 2015). Rates of hippocampal neurogenesis can be preserved throughout life if corticosterone levels are stabilized in middle age by adrenalectomy and low-level corticosterone replacement (Cameron and McKay, 1999). Furthermore, young plasma rejuvenates both cognition and hippocampal neurogenesis in aged mice, potentially through the activity of eotaxin, β2 macroglobulin, growth factor differentiation 11 or an as yet, unidentified factor (Mendelsohn and Larrick, 2011; Villeda et al., 2011, 2014). Memory-impaired aging rats have elevated levels of serum Gro-KC and RANTES and hippocampal GM-CSF relative to elite agers (Scheinert et al., 2015) and long-term daily exercise can rejuvenate hippocampal neurogenesis and memory scores while modulating some systemic and central cytokines (Speisman et al., 2013a). Note that not all systemic factors produce coordinated effects on neurogenesis across brain regions. For example, systemic growth hormone stimulates hippocampal but not subependymal neurogenesis (David Aberg et al., 2010). Given their effects on cytokines, HPA axis activity, vascular fitness and growth factor levels in the brain, indomethacin and rosiglitazone could rejuvenate hippocampal and subependymal neurogenesis by modulating these or other factors (Stubbs et al., 2002; Gerber and Bale, 2012). We are currently exploring their effects on circulating and central cytokine levels.

A growing body of literature has revealed that relationships between microglia, neurogenesis and cognition are complex (Ekdahl et al., 2009; Schwartz et al., 2013). Consistent with published work, we not only found increased microglia numbers in middle-aged and aged rats (Mouton et al., 2002) but found reduced proportions of resting and increased proportions of activated and/or phagocytic microglia in aging rats with a robust increase in phagocytic microglia in aged rats (Perry et al., 1993; Frank et al., 2006; Ziv et al., 2006). Previous work has shown that PPAR-γ activation suppresses microglial recruitment and reduces age-related increases in inflammatory signaling (Jiang et al., 1998; Dannhardt and Kiefer, 2001; Cowley et al., 2012). Longer treatment courses may been required to see effects of rosiglitazone and indomethacin on microglial activation in the current study. Interestingly, in young rats, higher activated microglia numbers related to better memory scores while higher phagocytic microglia numbers related to poorer memory scores. While ‘activated’ microglia could mediate some beneficial on synaptic pruning and maturation (Paolicelli et al., 2011; Tremblay et al., 2011), it is important to note that the number of microglia expressing markers of activation and/or phagocytosis was quite low in young rats. Our finding that resting microglia numbers correlated negatively with memory scores in aged rats most likely reflects dysregulated microglial immune signaling (Norden and Godbout, 2013).

In summary, relatively short-term indomethacin and rosiglitazone treatment improved the memory of aging rats after retraining, and increased new hippocampal neuron numbers in aging rats and new subependymal zone neuroblasts/neurons in all rats. Rosiglitazone treatment also increased the proportion of resting microglia in aging rats, which could modulate both neurogenesis and cognition. We expect that longer treatment and survival times may reveal more robust effects of these drugs and stronger relationships between indomethacin- and rosiglitazone-rejuvenated neurogenesis and cognition. New neuron numbers in the hippocampus and subependymal zone of rats were generally unrelated, suggesting that NSAIDs may work through region-dependent mechanisms to rejuvenate neurogenesis, which is unsurprising given the number of targets that they modulate. Overall our data suggest the feasibility of investigating longer-term and perhaps more specific immunomodulatory strategies for age-related cognitive decline.

## Author contributions

BO, RS, and TF designed the experiments, JM, RS, AA, VS, CL, AR, AK, TF, and BO collected behavioral data, processed samples and collected histological data, BO, RS, and JM analyzed the data and wrote the manuscript and all authors read and edited the final document.

### Conflict of interest statement

The authors declare that the research was conducted in the absence of any commercial or financial relationships that could be construed as a potential conflict of interest.
